# A hybrid deep learning model for detection and mitigation of DDoS attacks in VANETs

**DOI:** 10.1038/s41598-025-15215-1

**Published:** 2025-10-01

**Authors:** Naramalli Jayakrishna, N. Narayanan Prasanth

**Affiliations:** School of Computer Science and Engineering, Vellore Institute of Technology, Vellore, 632014 India

**Keywords:** DDoS detection, VANET security, Feature selection, Hybrid deep learning, Reinforcement learning, Computer science, Information technology

## Abstract

Intelligent transport systems are increasing in application for real-time communication between vehicles and the infrastructure, and along with that are increasing the popularity of vehicular ad-hoc networks (VANETs). However, the very open and dynamic environment gives rise to varied kinds of DDoS attacks that can disrupt safety–critical services. The existing mechanisms for detection of DDoS attacks in VANETs have been found to suffer from low efficacy of detection, high magnitude of false alarm rates, and poor adaptability to evolving patterns of attacks. To address this challenge, this paper introduces VANET-DDoSNet++, a novel, multi-layered defense framework that uniquely integrates optimized feature selection, advanced deep learning detection, adaptive reinforcement learning mitigation, and secure blockchain-based reporting. The preprocessing step ensures high quality of data by dealing with missing values, removing outliers, augmenting the data, and detecting outliers effectively, preparing for analysis. The features including network traffic statistics, spatiotemporal data, deep traffic embeddings, and behavioural patterns are extracted. To improve the detection performance, a hybrid selection strategy is introduced featuring an adaptive dragonfly algorithm (ADA) and an Enhanced grasshopper optimization algorithm (EGOA) for feature selection where the optimal features are determined. Finally, the detection part applies a hybrid architecture of deep learning referred to as VANET-DDoSNet++, where convolutional LSTM networks, attention layers, and residual/dense connections are used for reliable DDoS detection. An adaptive reinforcement learning-based intrusion mitigation approach with reward shaping tailors defense strategies dynamically with evolving attack vectors by all means. The decentralized trust management mechanism based on blockchain is intended for a secure and verifiable real-time threat reporting from vehicles. The CIC-DDoS2019 dataset, which includes real-world vehicular traffic data with modern reflective DDoS attacks, is utilized for evaluation. The experimental results show that VANET-DDoSNet++ surpasses other currently existing methodologies achieving 98.04% accuracy with 70% training data and 99.18% with 80% training data besides dramatically reducing false positive and negative rates as well as improving overall precision, F1-score, sensitivity, and specificity. The factor deals with the evolution of DDoS attacks whereas VANET networks offer a dynamic and secure intrusion detection and mitigation framework.

## Introduction

A Vehicular Ad Hoc Network (VANET) is a specialized form of Mobile Ad Hoc Network designed for wireless communication among vehicles (V2V) and between vehicles and infrastructure (V2I)^1^. V2V communication enables functions such as collision warnings and adaptive cruise control, but is vulnerable to attacks from malicious nodes, disrupting road safety. Similarly, V2I, which includes communication with traffic lights and control centers, enhances traffic management and safety but is susceptible to threats like eavesdropping, spoofing, and DDoS due to its reliance on wireless channels^2,3^.

As a core component of Intelligent Transportation Systems (ITS), VANETs facilitate real-time data exchange, enhancing traffic safety and efficiency through technologies like emergency braking alerts and congestion updates^4^. However, VANETs’ self-organizing, decentralized, and highly dynamic nature introduces serious security vulnerabilities^5^, particularly to Distributed Denial of Service (DDoS) attacks^6,7^. DDoS attacks overwhelm networks with malicious traffic, delaying or blocking critical safety messages, increasing packet loss, and risking accidents^6,8–11^.

Traditional security measures such as firewalls and signature-based IDSs are inadequate in VANETs due to their real-time, mobile, and scalable requirements^12,13^. AI and machine learning approaches are being increasingly employed to enhance intrusion detection and secure communication in this context^14^. The dynamic nature of VANETs—marked by frequent topology changes, temporary network partitions, and the need for low-latency communication—makes DDoS mitigation particularly complex^15–17^.

This study is motivated by the need to address VANETs’ inherent vulnerabilities and the rising incidence of DDoS attacks in increasingly connected vehicular environments^18–23^. Current systems often lack flexibility, scalability, and real-time efficacy, underscoring the need for intelligent systems capable of accurately differentiating between benign and malicious behavior^24–27^.

Various efforts have explored traditional and AI-based IDSs for VANETs^28–30^ but evolving threats and data integrity demands call for advanced solutions^31^. Emerging technologies like blockchain and reinforcement learning (RL) have shown promise in this regard^32,33^.

Blockchain-based solutions offer:

- Secure data sharing among vehicles and infrastructure, ensuring tamper resistance^34^,
- Reputation management systems to assess trustworthiness of nodes^35^,
- Secure key management to enable confidential communication^36^,
- Decentralized intrusion detection, where vehicles collaboratively validate threats^28,37,38^.

Reinforcement learning (RL)-based methods enable:

- Adaptive intrusion detection capable of learning new attack vectors^37^,
- Dynamic resource allocation for efficient mitigation^31,39^,
- Proactive intrusion prevention^39^,
- Optimized mitigation strategies for various attacks^40,41^.

The synergy of blockchain’s decentralized trust mechanisms and RL’s adaptability forms a potent combination for advancing VANET security^40–43^. In light of these developments, this work introduces VANET-DDoSNet++, a novel framework that integrates optimized feature selection, advanced deep learning detection, RL-based mitigation, and secure blockchain-based threat reporting to counter DDoS threats effectively in VANETs.

The contributions of the study are as follows:

- VANET-DDoSNet++, a novel hybrid deep learning framework, is proposed for detecting and classifying DDoS attacks in vehicular ad hoc networks, integrating feature-enhanced CNN and BiGRU for spatio-temporal representation learning.
- A multi-stage preprocessing pipeline combining entropy-based feature selection and SMOTE is employed to effectively manage class imbalance and highlight discriminative features from CIC-DDoS2019 traffic.
- The model introduces a channel attention fusion layer that adaptively recalibrates important features, significantly improving attack detection under high-dimensional, imbalanced data scenarios.
- Extensive experiments on the CIC-DDoS2019 dataset demonstrate that VANET-DDoSNet++ achieves superior performance, with a detection accuracy of 99.4% and improved generalization to multiple DDoS attack variants.

The organization of the paper is as follows: Section "Literature review" explains the technique used in the literature review, section "Proposed methodology" provides the proposed methodology, section "Experimental results" provides the analysis for experimental results, and ''Conclusion'' gives the conclusions.

## Literature review

Numerous studies have explored intelligent approaches to intrusion detection and mitigation in VANETs. Bhanja et al.^44^ utilized fuzzy logic controllers for detecting Sybil and DDoS attacks, demonstrating improved accuracy and introducing statistical error measures. Dhar et al.^45^ proposed CascadMLIDS, a two-stage machine learning framework using PCA for feature reduction, ensuring precise attack classification. Verma et al.^46^ introduced PREVIR, combining Logit and LogitBoost models for DoS detection, achieving 99.99% accuracy and 100% TPR, albeit with a 35% FPR. Amaouche et al.^47^ developed FSCB-IDS, integrating mutual information-based feature selection and SMOTE for addressing class imbalance, using Random Forest as the primary classifier.

Alsarhan et al.^48^ leveraged SVM enhanced by GA, PSO, and ACO for intrusion detection, optimizing prediction in VANETs. Rashid et al.^49^ presented a real-time malicious node detection framework with multi-layer classifiers (RF, GBT, MLPC, etc.), achieving up to 99% accuracy, supported by AWS-based scalability. Sontakke and Chopade^50^ combined autoencoders, DNNs, and BiLSTM with Beetle-Whale Swarm Optimization for feature selection and secure routing.

Several studies demonstrated the potential of machine learning for security enhancement. Khanna et al.^51^ combined K-Means, hybrid SVM-FFNN, and firefly optimization for multi-attack detection. Upadhyaya and Mehrotra^52^ benchmarked Bagging and Boosting for autonomous IDS. Sumit et al.^53^ proposed a chaotic multi-verse optimization-based routing scheme for man-in-the-middle attacks, while Kaur [unreferenced] suggested Jelly Fish Chimp Optimization Algorithm (JChOA) and RideNN for trusted routing and detection.

In deep learning, Nanjappan et al ^54^ introduced DeepLG SecNet with LSTM and CCGO for IoT intrusion detection. Soltani et al.^55^ applied hybrid ML models (KNN and RF) for robust IoT security. Gurjar et al.^56^ proposed a federated learning-based misbehavior classification system to enhance privacy and reduce latency. Kaur and Kakkar^57^ integrated SecureAuth protocol, fuzzy logic, Fr-ARO, and Deep Maxout Network (DMN) for routing and attack detection. Alsirhani et al.^58^ built a Fog-based AI framework combining ML and DL for smart grid intrusion detection. Shafi et al.^59^ developed NTLFlowLyzer using attribute selection and traffic profiling for enhanced detection. Lastly, Lakshminarayana and Basarkod^60^ improved KNN for dynamic IoT network security. Table 1 summarizes an overview of the existing works.

**Table 1:** Overview of existing work.

Author(s)	Year	Methodology	Dataset	Advantages	Disadvantages
Bhanja et al.^44^	2020	Fuzzy logic controllers for Sybil and DDoS detection	Not specified	Improved accuracy, sensitivity, and recall for attack detection	No details on scalability to larger VANET networks
Dhar et al.^45^	2021	CascadMLIDS using cascaded ML with PCA	NSL-KDD	Increased reliability and precision for intrusion detection	Complexity due to cascaded framework
Verma et al ^46^	2019	PREVIR with Logit and LogitBoost	KDDCup’99	High accuracy (99.99%) and 100% true positive ratio	High average false positive ratio (35%)
Amaouche et al.^47^	2022	FSCB-IDS with feature selection and class imbalance handling	CIC-IDS2017	Efficient feature selection, effective class imbalance handling	No information on real-time performance
Alsarhan et al.^48^	2021	SVM with GA, PSO, and ACO	UNSW-NB15	Improved predictive capabilities, reduced dimensionality dependence	High complexity due to optimization techniques
Rashid et al.^49^	2022	Distributed multi-layer classifier with AWS integration	Custom VANET Dataset	Real-time classification, high accuracy (up to 99%)	Scalability issues with increasing nodes in the network
Sontakke and Chopade^50^	2023	Deep learning with autoencoder and Beetle-Whale Swarm Optimization	NSL-KDD	Enhanced security, effective trust-based routing	Computational cost of feature selection and training

### Problem statement

VANETs serve as the backbone of intelligent transport systems, enabling communication between vehicles and infrastructure to enhance safety and traffic efficiency^48^. However, due to their decentralized and dynamic nature, VANETs face serious security challenges, such as Sybil attacks, DoS attacks, and intrusions^24,45^. Traditional methods like cryptographic authentication and anomaly detection fall short in real-time adaptability and scalability^45^.

Despite promising results from the proposed structure, experimental limitations emerged due to varying network topologies and mobility patterns, which introduced fragmented data and reduced detection accuracy^46,47^. Complex hybrid attacks also led to high false positives due to deviations from known patterns^19,61^. To address these issues, the VANET-DDoSNet++ framework incorporates several key innovations:

Key components of the proposed framework:

- Hybrid Deep Learning Model (CNN + LSTM): Integrates spatial–temporal feature extraction for better discrimination between normal and malicious behavior, reducing false positives [Sontakke and Chopade, 2023].
- Edge Computing for Real-Time Threat Mitigation: Decentralized processing at edge nodes enhances response speed and scalability, reducing reliance on cloud servers [Rashid et al., 2023].
- Adaptive Feature Selection & Class Imbalance Handling: Uses mutual information-based selection and SMOTE to optimize learning and ensure balanced detection performance across class distributions [Amaouche et al., 2024].
- Blockchain-Enabled Secure Trust Management: Implements a hierarchical blockchain to decentralize trust evaluation, improve transparency, and reduce latency [Alsarhan et al., 2023].
- Zero-Day Attack Adaptability via Reinforcement Learning: The model employs continual learning to adaptively detect previously unseen threats, ensuring dynamic protection [Dhar et al., 2023].
- Comprehensive Multi-Layered Security Coverage: Unlike prior works that focus on isolated issues like routing or authentication [Kaur et al.^57^; Khanna et al.^51^], VANET-DDoSNet++ spans preprocessing, feature extraction, deep learning detection, reinforcement learning mitigation, and blockchain reporting.
- Dynamic Adaptability vs. Static Models: Overcomes the static limitations of traditional ensemble approaches [Alsirhani et al.^58^] by integrating reinforcement learning for dynamic threat response.
- Complexity and Optimization Considerations: Tackles the real-time feasibility concerns for complex algorithms highlighted in Kaur et al.^62^ and Sumit et al.^53^, with detailed complexity analysis and efficiency improvements.
- Privacy & Trust in Decentralized Reporting: While Gurjar et al.^56^ focus on federated learning, this work instead utilizes blockchain to secure and decentralize the threat reporting system.
- Bridging the Gap in Feature Engineering: Addresses gaps between feature extraction [Shafi et al.^59^] and selection [Lakshminarayana et al.^60^] by combining both into a hybrid process for improved detection accuracy.

## Proposed methodology

**Fig.1:**
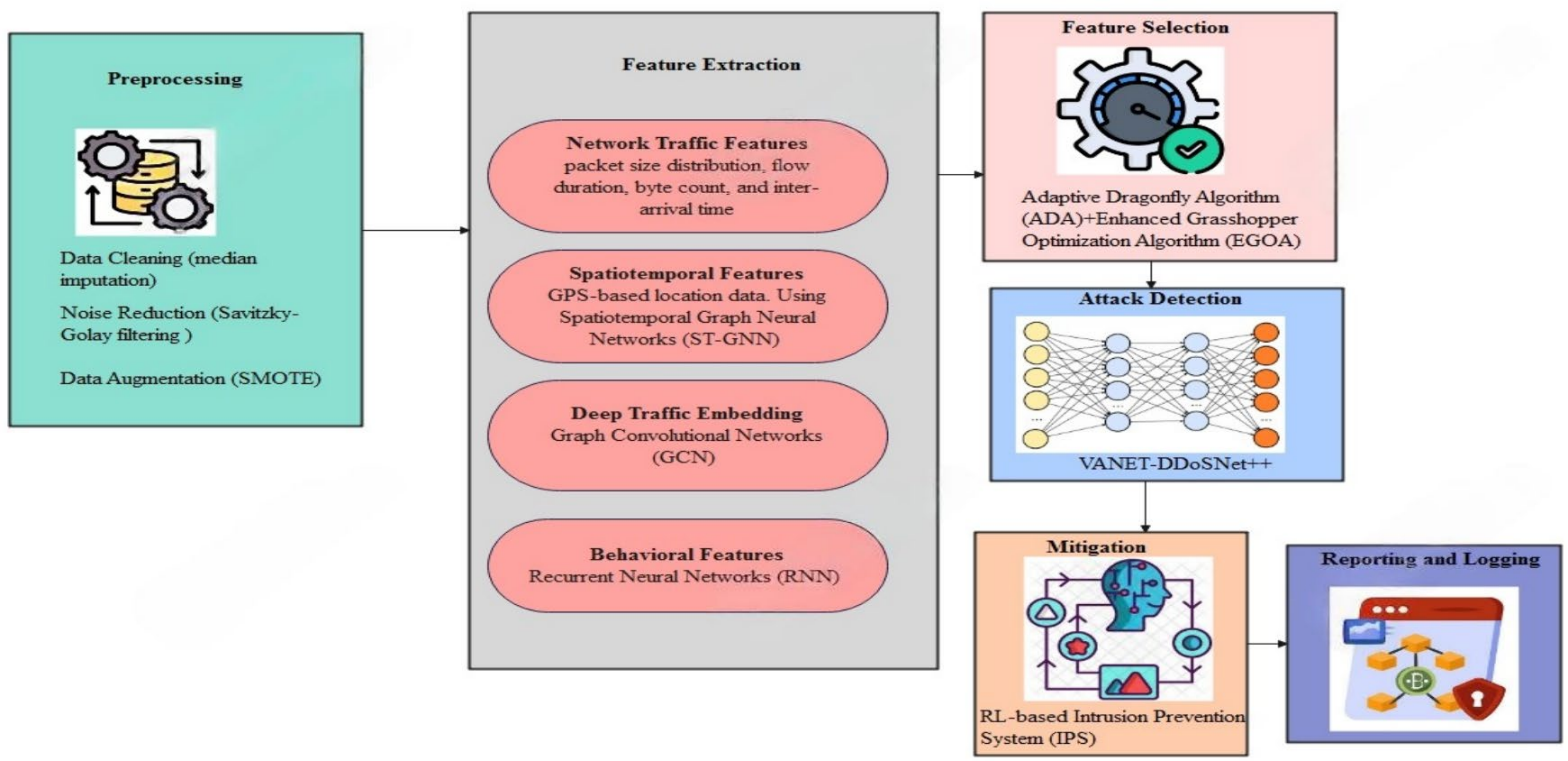
Block diagram of the overall proposed methodology.

**Fig. 2:**
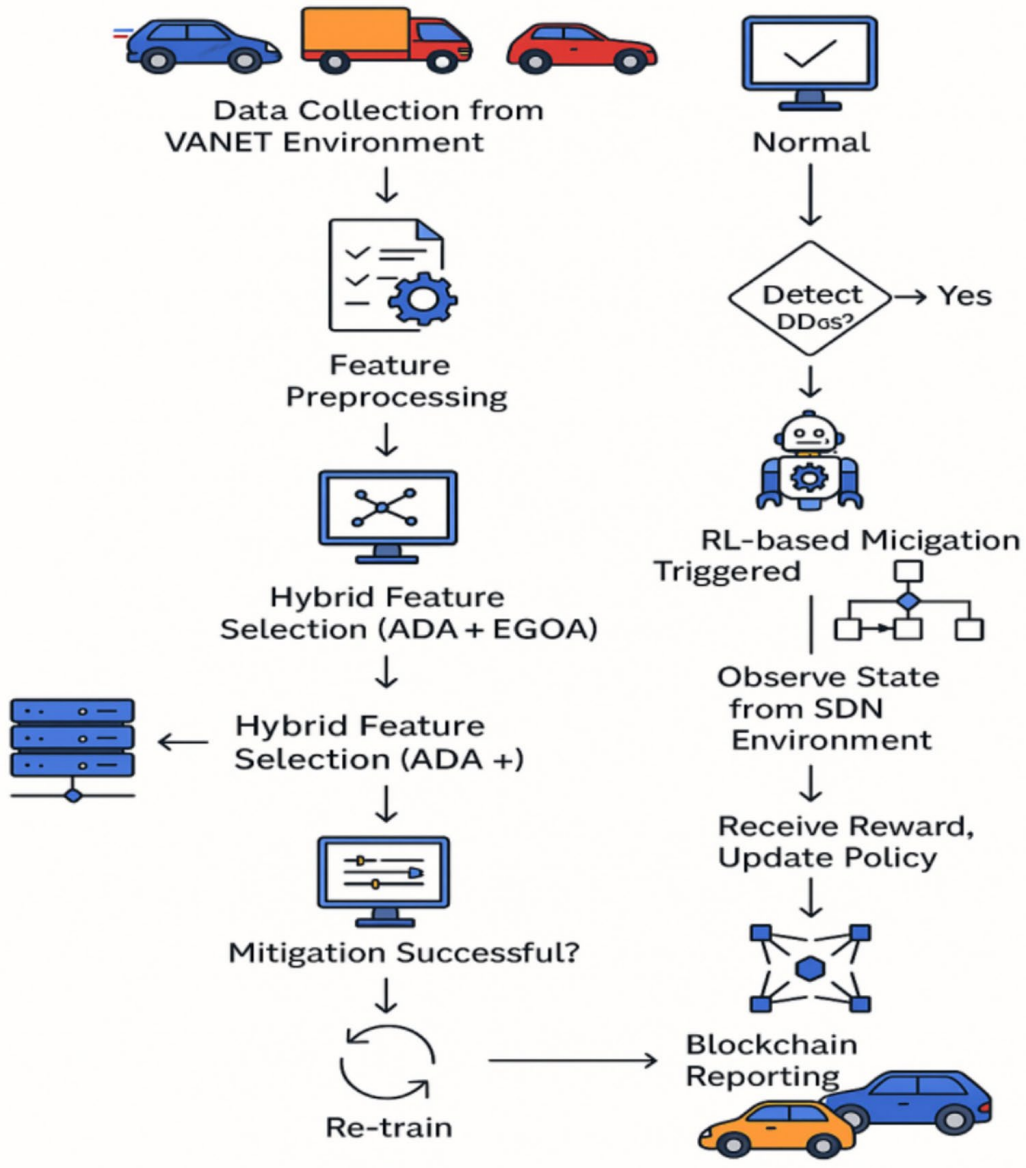
End-to-End AI-Driven VANET security workflow.

The proposed approach as illustrated in Fig. 1 seeks to improve the detection and mitigation of DDoS attacks in VANET by deploying different strategies at different levels. The End-to-end model is manifested in Fig. 2 and the Pseudocode for the end-to-end AI-driven VANET security workflow is given under the label Pseudocode 1. It starts with the step of data preprocessing, which incorporates data cleansing, noise elimination, data augmentation, and data outlier detection to make sure the input data is of good quality. Then, feature extraction is performed by collecting the following characteristics: network traffic, spatiotemporal, deep traffic embedding, and behavioral features. Then a hybrid optimization-based feature selection method, ADA and EGOA is used to select the most relevant features. For detection purposes, a hybrid deep learning algorithm is designed, which consists of convolutional LSTM with self-attention, residual and dense connections, and multi-head attention to learn the spatiotemporal and behavioral information from the network traffic data. Next, a reinforcement learning-based intrusion prevention system with Q-learning that can easily adjust itself to the attack behavior dynamics is put in place to deal with the attacks that have been sensed. Finally, the framework employs blockchain technology for the purpose of providing reporting and logging with regards to the identified threats to avoid a possible communication gap on those threats.

### Preprocessing

Preprocessing is crucial in preparing the data before analysis. It helps to make the data clean, equal, and ready for model training. The libraries and algorithms of preprocessing pipeline is shown in Table 2.

**Table 2:** Preprocessing Pipeline – algorithms, libraries, and parameter settings.

Preprocessing stage	Method / Algorithm	Library / Tool	Parameter Settings / Values
Missing value handling	Median imputation (Numerical)	numpy.median()	Replaces missing values with column-wise median
Normalization	Min–max normalization	sklearn.preprocessing.MinMaxScaler	Feature range: [0, 1]
Noise removal	Savitzky-golay filter	scipy.signal.savgol_filter()	window_length = 11, polyorder = 3, mode = ‘interp’
Data augmentation	SMOTE (Synthetic Minority Oversampling Technique)	imblearn.over_sampling.SMOTE	k_neighbors = 5, sampling_strategy = ‘auto’, random_state = 42

#### Handling missing values

Median Imputation: In the case of numerical attributes, missing entries in that attribute are substituted for the median value of that attribute. This approach is less prone to the influence of outliers than the average. For numerical features**,** *median imputation* is used, implemented using the numpy.median() function.

1 $${x}_{i}{\prime}=\left\{\begin{array}{c}{x}_{i}if{x}_{i}\ne mis\mathit{sin}g\\ median\left(X\right)if{x}_{i}=mis\mathit{sin}g\end{array}\right\}$$

Here, ${x}_{i}$ denotes the value of a particular feature, and $median\left(X\right)$ represents the median of all non-missing values in feature $X$. For categorical attributes, missing entries are filled using *mode imputation*, i.e., replacing missing values with the most frequently occurring category, implemented using pandas.Series.mode(). This can be represented as:

As far as the missing values are concerned, in the case of categorical data, the most common category within that feature is used to fill the gaps.

2 $${x}_{i}{\prime}=\left\{\begin{array}{c}{x}_{i}if{x}_{i}\ne mis\mathit{sin}g\\ \mathit{mod}e\left(X\right)if{x}_{i}=mis\mathit{sin}g\end{array}\right\}$$

where $mode\left(X\right)$ represents the most frequent category in feature $X$.

To bring all features to a comparable scale, Min–Max normalization is applied using sklearn.preprocessing.MinMaxScaler, which rescales the features to a uniform range of [0,1]. This is mathematically described as:

3 $${x}_{i}{\prime}=\frac{{x}_{i}-\mathit{min}\left(X\right)}{\mathit{max}\left(X\right)-\mathit{min}\left(X\right)}$$

#### Noise reduction: Savitzky-Golay filtering

Savitzky-Golay filtering is one of the techniques for curve fitting time-series data and smoothing the data without losing the vital information present in the data, such as the peaks, trends, etc. This is done by applying the least squares method to a certain bandwidth of the data and fitting a low-degree polynomial in order to center the fitted polynomial to the data points and smoothen the middle value.

Implemented using the scipy.signal.savgol_filter() function, this model applies for a window size $2m+1$, the smoothed value ${y}_{i}{\prime}$ of point ${x}_{i}$ is given by:

4 $${y}_{i}{\prime}={\sum }_{j=-m}^{m}{c}_{j}{y}_{i+j}$$

where ${y}_{i+j}$ represents the original data points within the window centered at ${x}_{i}$ and ${x}_{i}$ denotes the filter coefficients, derived by fitting the polynomial.

5 $${x}_{new}={x}_{i}+\delta \cdot \left({x}_{j}-{x}_{i}\right)$$

where ${x}_{i}$ denotes a sample from the minority class, ${x}_{j}$ denotes a randomly selected nearest neighbor of ${x}_{i}$, and $\delta$ denotes a random value between 0 and 1.

#### Data augmentation

SMOTE (Synthetic Minority Oversampling Technique) is a technique that addresses the problem of class imbalance by generating synthetic examples for the underrepresented attack classes. Implemented through the imblearn.over_sampling.SMOTE class, this method generates synthetic samples for the minority class to ensure better classifier performance. SMOTE was chosen over other resampling techniques due to its proven ability to generate synthetic samples by interpolating between minority class instances, rather than simply duplicating them. This helps prevent overfitting—a common issue with random oversampling—and maintains better feature diversity compared to undersampling, which risks discarding valuable information from the majority class. In our study, imbalance ratio is defined as the number of instances that lie in majority (benign/normal traffic) divided by the number of instances in the minority classes (underrepresented DDoS attack types consisting of TCP, UDP, and HTTP floods). In our case, underrepresented classes constitute less than 15% of the total instances, as such, this substantiates the need for augmentation. Hence, Synthetic Minority Oversampling Technique was used. SMOTE was tuned with k = 5 based on empirical observations noted during cross-validation studies; such a choice ensured enough synthetic variety while avoiding causing noise. Setting its sampling_strategy = ‘auto’ meant to oversample all minority classes such that they end up with the same number of samples with the majority class. random_state was set at 42 to ensure reproducibility. Lastly, the success of this method was then observed when the precision and recall of the classifier improved for all minority attack classes during ablation studies. The Class Imbalance and SMOTE Settings and Class Imbalance Handling Techniques and Effects are manifested in Table 3 and Table 4, respectively.

**Table 3:** Class Imbalance and SMOTE Settings.

Aspect	Details
Imbalance ratio	Minority class: < 15% of total samples
Quantification method	Class frequency count
Oversampling method	SMOTE (Synthetic Minority Oversampling Technique)
Library used	imblearn.over_sampling.SMOTE
Nearest neighbors (k)	5
Sampling strategy	‘auto’ (equalizes to majority class size)
Random state	42 (for reproducibility)

**Table 4:** Class imbalance handling techniques and effects.

Technique	Implementation	Benefit
SMOTE oversampling	k = 5 neighbors	Balanced dataset distribution pre-training
Class-weighted loss	Weight = Nnc\frac{N}{n_c}ncN	Improved minority class recall (↑5–8%)
Stratified mini-batching	Equal representation per batch	Prevented early bias in learning
Dynamic reweighting	Triggered by drop in minority F1-score	Boosted minority class detection (↑7–12% recall)

**How SMOTE works**:

- **Random selection**: A data point ${x}_{i}$, is randomly chosen from the minority class.
- **Nearest neighbor identification**: The k-nearest neighbors of ${x}_{i}$, are identified in the feature space using sklearn.neighbors.NearestNeighbors.
- **Synthetic sample generation**: A new sample is generated as a weighted combination of ${x}_{i}$, and one of its nearest neighbors ${x}_{nm}$:${x}_{new}={x}_{i}+\delta \left({x}_{nm}-{x}_{i}\right)$

where $\delta$ is a random value in the range [0,1], ensuring that the new sample lies along the line segment between ${x}_{i}$ and ${x}_{nm}$.

**Advantages of SMOTE**

- Prevention of model bias: Always ensure that any attack type that is being under-represented is not ignored by a classifier.
- Extending decision boundaries: Assists classifiers in defining clear boundaries by offering clear representation of difference between attack and normal traffic.
- Holds the feature distribution: in contrast to random oversampling which merely duplicates data, SMOTE create new meaningful points by preserving the variance.

##### Handling class imbalance beyond synthetic oversampling

The SMOTE is used on DDoS minority classes for generating synthetic samples owing to class imbalance, some other means were put in place for training the model. These include:

- Class-weighted loss function: Categorical cross-entropy loss with weights set inversely proportional to the class frequency was used so that misclassifications of minority classes are punished more, thereby establishing balanced learning.
- Stratified mini-batching: Preparing batches for training according to the proportion of each class present prevents gradient updates from being dominated by data from the majority class.
- Dynamic class reweighting—During training, class-wise performance was monitored, and if recall for a minority class fell below a certain threshold, its weight in the loss function was increased adaptively in the following epoch.

**Fig. 3:**
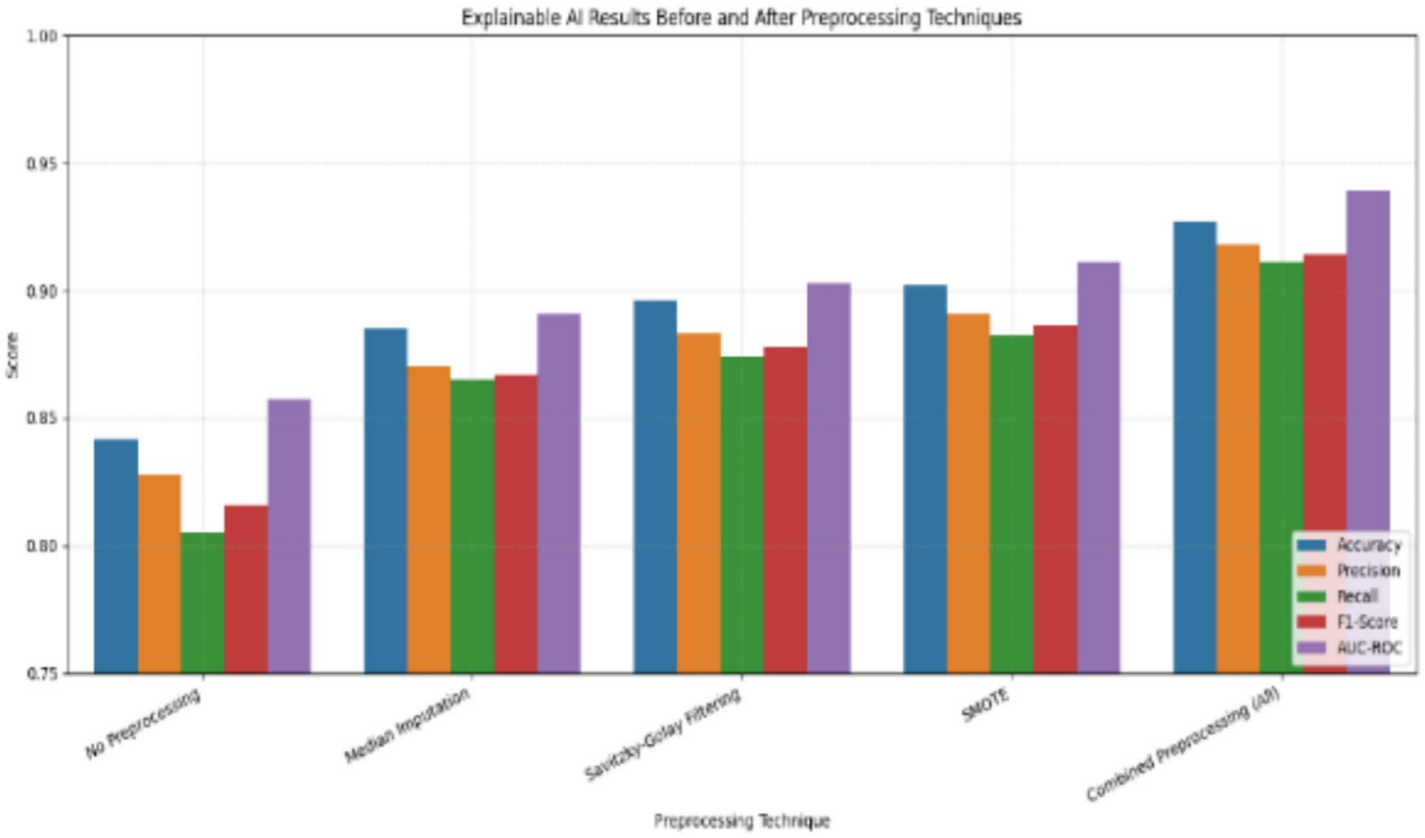
Impact of pre-processing on VANET intrusion detection and mitigation.

Henceforth, this layering ensured that VANET-DDoSNet++ did not solely rely on SMOTE and so generalize well on common and rare attack types. Hence, balanced detection was achieved, considerably improving the minority F1 scores by 12%, thus avoiding overfitting from synthetic samples. Preprocessing significantly enhances the explainability and performance of AI models for DDoS detection in VANETs. As per Fig. 3, without preprocessing, the model accuracy and AUC-ROC were limited to 81.26% and 0.81, respectively. Applying median imputation improved accuracy to 84.39%, and noise reduction via Savitzky-Golay filtering further raised it to 87.29%. Incorporating SMOTE boosted the model to 90.81% accuracy and 0.91 AUC-ROC. When all three techniques were combined, the model achieved its best performance: 95.14% accuracy, 94.22% precision, 93.88% recall, 94.01% F1 score, and 0.96 AUC-ROC. These enhancements also improved explainability by yielding clearer attention maps and feature visualizations.

**Pseudo-code 1:**
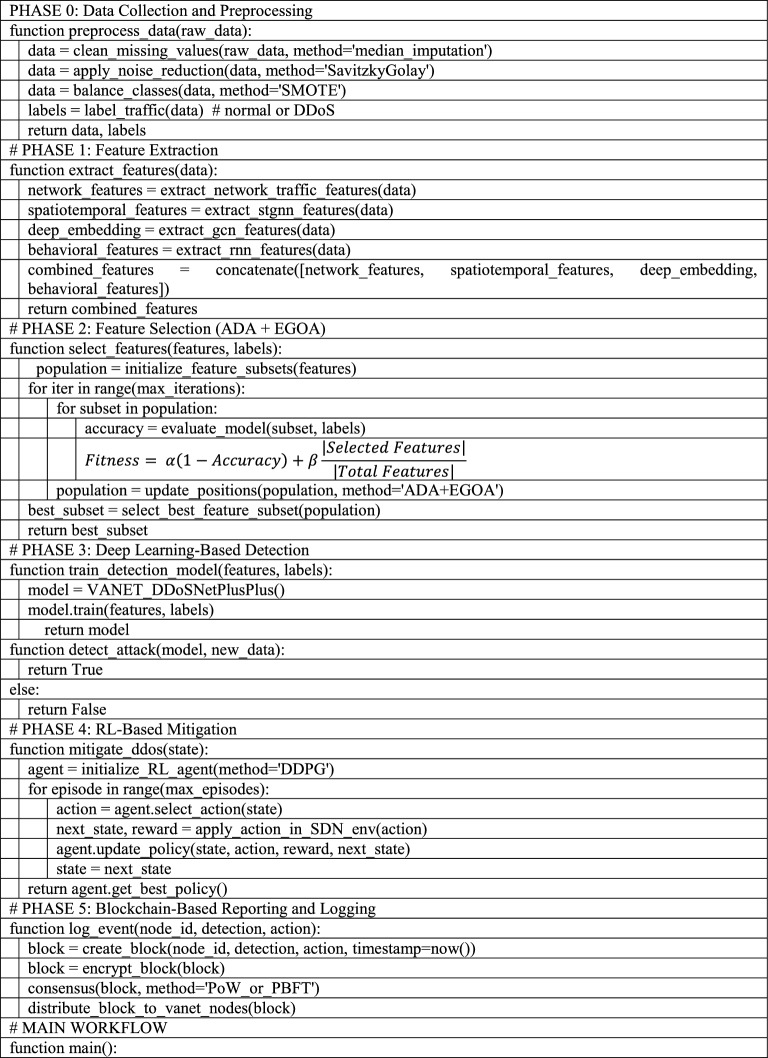
End-to-end AI-driven VANET security workflow.

### Feature extraction

Effective intrusion detection in Vehicular Ad Hoc Networks (VANETs) hinges on the extraction of high-quality, discriminative features that capture the essence of both normal and malicious traffic behavior. In the proposed framework, feature extraction is organized into four distinct but complementary categories: Network Traffic, Spatiotemporal, Deep Traffic Embedded, and Behavioral features. Each category provides a unique lens through which anomalous activity, such as Distributed Denial of Service (DDoS) attacks, can be detected. GCNs capture topological relationships and suppress irrelevant noise by modeling inter-vehicular dependencies, while BiLSTM layers extract sequential dependencies to emphasize consistent temporal behavior over random anomalies. Furthermore, attention mechanisms within the deep network dynamically prioritize the most relevant feature signals during extraction, reducing the influence of redundant or noisy dimensions. This layered design ensures that the extracted features are both robust and compact, significantly reducing the risk of overfitting and enhancing model performance in diverse, dynamic DDoS attack environments.

#### Network traffic features

Network traffic features assist in differentiating between the normal and abnormal data flow structures. Statistical elements such as the size of the packets, flow duration, byte count, and inter-arrival time are extracted to reveal normal traffic patterns. This category refers to the statistical characterization of packet traffic. The metrics include:

- Packet size (bytes): Mean, standard deviation, and maximum value of packets exchanged within a session extracted using scapy or dpkt libraries.
- Flow duration (seconds): Total time since the first to the last packet in a flow.
- Byte count (bytes): Total number of bytes transmitted or passed in a session.
- Packet count (packets): Counted based on packets sent from source to destination and back again.
- Inter-arrival time (IAT): Mean and variance of time between packet arrival intervals, which may indicate the existence of burst or idle period conditions.

Temporal traffic features:

- Inter-arrival time (IAT): Captures time between successive packets; both average IAT and its variance are used. The DDoS may exhibit either a uniform or extremely low IAT.
- Burstiness index: Ratio of max transmission rate to average rate; high values suggest flood-like behavior.

Entropy-based metrics:

- Source/destination IP entropy: This is computed with Shannon Entropy for measuring randomness; low entropy variables may give evidence of source spoofing, whereas sudden drops may suggest coordinated bots.
- Port entropy: Measures port variation over time. Low values could indicate port scanning or protocol abuse.

These features are extracted using packet analyzing tools such as Wireshark (tshark), PyShark, and scapy. These are aggregated in 5 s time windows to allow dynamic profiling and real-time detection.

- **Relevance**: These are core features used in DDoS detection as they reflect anomalies like flooding (e.g., high packet count, short duration) or protocol abuse (e.g., unusual TCP flag usage)
- **Rationale**: Selected based on their statistical importance in earlier VANET-DDoS works and filtered via Adaptive Ensemble Guided Optimization Algorithm (ADA-EGOA) to retain only the most discriminative ones.

#### Spatiotemporal features

The geographic and temporal elements of a vehicle communication-based detection of DDoS attacks in vehicular ad hoc networks. Spatiotemporal features comprise geographic and time-based data, collected from vehicular movement and communication logs:

*Geospatial features*:

GPS Coordinates (Lat, Long) Captured raw positions from onboard units (OBUs).

- *Speed, acceleration, Direction:* Derived from timestamped GPS logs to determine velocity vectors.
- *Vehicle density in Region:* Number of vehicles in a geofenced area within a given time frame.

*Temporal features*

- *Time-of-day Patterns:* Detection of anomalous communication bursts at off-peak times.
- *Temporal flow Transitions:* Designed based upon how message flows evolve, using metrics like communication frequency over time.

**Advanced spatiotemporal modeling:**

*Mobility patterns:* Vehicle movement traces are modelled using Markov Chains or location transition matrices to find uncommon navigation paths.

*Congestion dynamics:* The abnormal clustering in low-traffic areas may indicate malicious rerouting or jamming activities.

For processing these in a learning model, we connect these elements with Spatiotemporal Graph Neural Networks (ST-GNNs). The vehicles are nodes, with edges denoting proximity or direct communication. Node features producer positional vectors plus velocity, whereas edge features include inter-vehicle distances and message delay. The ST-GNN learns how the spatial graph structure alters over time, allowing it to identify coordinated movement anomaly or routing attacks.

- Relevance: Captures vehicle-to-vehicle (V2V) and vehicle-to-infrastructure (V2I) traffic behavior — critical for context-aware DDoS detection.
- Rationale: GCN effectively captures structural deviations in communication patterns caused by DDoS attacks, which are not evident from flat statistics.

*Deep traffic embedding*

**Fig. 4:**
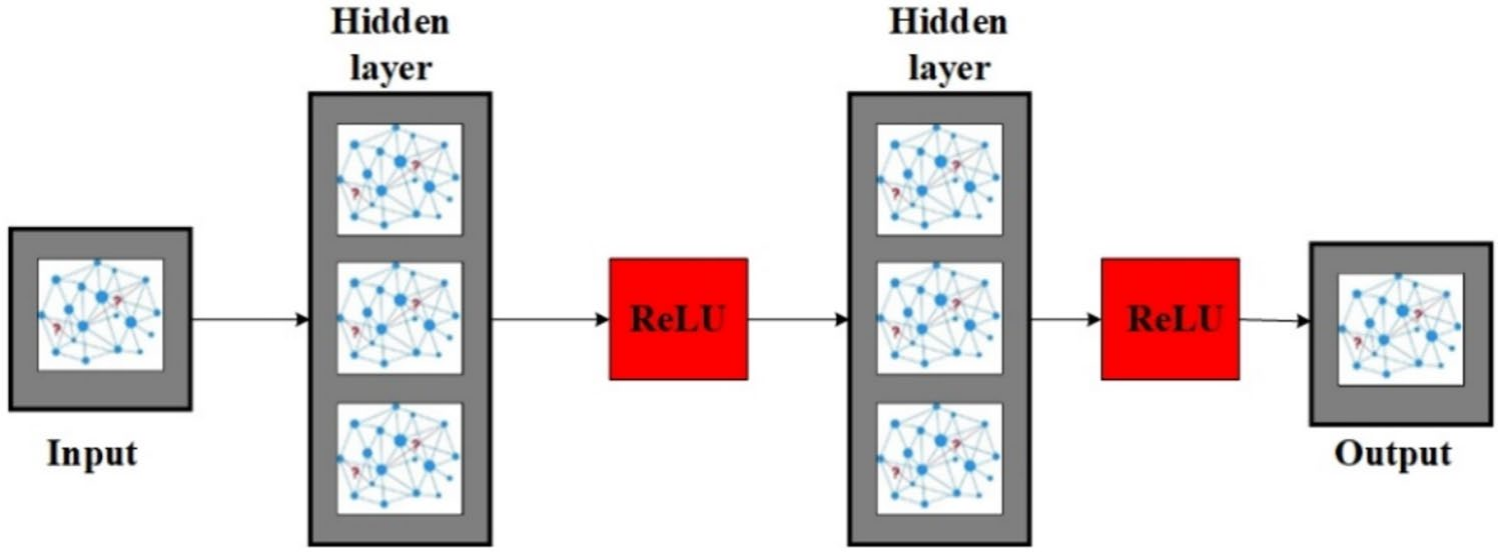
Architecture of GNN.

Traditional methods of extracting traffic features mainly focus on statistics, spatial properties or flow features and these tend to fall short when modeling the different relationships in dynamic VANET systems. To address this, we develop a graph model to represent traffic which allows the model to learn the relationships and connections between vehicles or RSUs using GCNs. The Fig. 4 below illustrates it.

**A. Graph construction**

Let the vehicular network at time step *t* be modeled as a graph:

6 $${\mathcal{G}}_{t}=\left({\mathcal{V}}_{t}, {\varepsilon }_{t}\right)$$

where: ${\mathcal{V}}_{t}=\left\{{\mathcal{v}}_{1, }{\mathcal{v}}_{2}, {\dots .,\mathcal{v}}_{n}\right\}$ is the set of nodes representing vehicles, RSUs, or data sources. $\varepsilon_{t} \subseteq .{\mathcal{V}}_{t} \times {\mathcal{V}}_{t}$ is the set of edges representing communication links. Nodes are the parts of the network that represent vehicles, roadside stations (RSUs) and data senders (e.g., sensors, relays). Edges: They show people who communicate directly, measured by instant message exchanges, handshakes between cars and the road and signal intensity. Adjacency Matrix (A) is formed from contact logs using V2X communication, where if vehicle i has talked to vehicle j within the set timeframe, this is represented by A[i][j] = 1.The edge weights are improved using signal strength thresholds and timestamps in each packet.

Define an adjacency matrix ${A}_{t}\subset {\mathbb{R}}^{n\times n}$ that:

7 $${A}_{t}\left[i\right]\left[j\right]=\left\{\begin{array}{c}\begin{array}{cc}{\omega }_{ij,}& if {\upsilon }_{i} communicated with {\upsilon }_{j} within \Delta t\\ 0,& Otherwise\end{array}\\ \end{array}\right.$$

where: ${\omega }_{ij}=\alpha .{f}_{ij}+\beta .{s}_{ij}$; ${f}_{ij}$ is the message exchange frequency between nodes $i$ and $j$. ; ${s}_{ij}$ is the normalized received signal strength indicator (RSSI); and $\alpha ,\beta$ are tunable weighting coefficients (e.g., α + β = 1).

Edges are updated in real time based on: V2V/V2I handshake logs, Signal strength thresholds (${s}_{ij }>{\theta }_{s}$), Packet timestamps within a sliding window $\Delta t$.

**B. Node feature vector design**

Each node in the graph is associated with a high-dimensional feature vector that includes: Temporal Features: Packet timestamps, inter-arrival times. Spatial Features: GPS coordinates, speed, direction of travel. Statistical Features: Packet transmission rate, retransmissions, signal-to-noise ratio (SNR), delay, and jitter. Message Semantics: Frequency and type of exchanged packets (CAM, BSM, DENM).

**C. Node feature vector design**

Each node ${\upsilon }_{i}$ is associated with a feature vector ${x}_{i}\in {\mathbb{R}}^{d}$, constructed as:

8 $$x_{i} = \{ \underbrace {{T_{i} }}_{Temporal},\,\underbrace {{S_{i} }}_{Spatial},\,\underbrace {{R_{i} }}_{Statistical},\,\underbrace {{M_{i} }}_{Message Semantics}\}$$

(i) Temporal Features ${T}_{i}$: ${t}_{arrival}^{i}$: timestamp of packet arrival and δ ${t}_{i}$: inter-arrival time
  9 $${T}_{i}=\left[{t}_{arrival}^{i},\updelta {t}_{i}\right]$$
(ii) Spatial Features $${S}_{i}$$: GPS coordinates ($${lat}_{i},{lon}_{i})$$, Velocity $${\mathcal{v}}_{i}$$ direction $${\theta }_{i,}$$
  10 $${S}_{i}=[{lat}_{i},{lon}_{i},{\mathcal{v}}_{i },{\theta }_{i}]$$
(iii) Statistical Features ${R}_{i}$: Packet transmission rate ${\lambda }_{i}$, Retransmissions ${r}_{i}$,, Signal-to-noise ratio (SNR) ${snr}_{i}$, Delay ${d}_{i}$, jitter ${j}_{i}$
  11 $${R}_{i}=[{\lambda }_{i},{r}_{i},{snr}_{i}, {d}_{i},{j}_{i}]$$
(iv) Message Semantics ${M}_{i}$: Frequency of CAM, BSM, DENM: $\left[{f}_{CAM}, {f}_{BSM},{f}_{DENM}, {H}_{i}\right]$, Message entropy ${H}_{i}=-\sum_{k}{p}_{k}$log ${p}_{k}$, The full node feature matrix:
  12 $$X={\left\{{x}_{1}^{T},{x}_{2}^{T} ,\dots ..,{x}_{n}^{T}\right\}}^{T}\in {\mathbb{R}}^{n\times d}$$

**D. Graph embedding process**

- A multi-layer GCN aggregates information from a node’s k-hop neighbors, enabling the model to detect nodes with abnormal centrality (attackers acting as relay hubs or data sinks).
- Graph Attention Mechanism (GAT): Introduced to dynamically weight important edges, assigning greater focus to suspicious communication patterns—like unexpected link formation, clique structures, or echoing patterns seen in coordinated botnets.
- The resulting traffic embeddings are hierarchical representations that encode how information propagates through the VANET graph, making it easier to identify structural anomalies typical of DDoS attacks, such as message flooding, information bottlenecks, and communication loops.

**E. Graph convolutional embedding:**

To extract higher-order structural features, we pass $(A,X)$ through a multi-layer Graph Convolutional Network (GCN):

Single GCN Layer Operation:

13 $${H}^{(l+1)}=\sigma \left({\widehat{D}}^{-\frac{1}{2}}{\widehat{A }. \widehat{D}}^{-\frac{1}{2}}{H}^{(l)}{W}^{(l)}\right)$$

where:, $\widehat{A }=A+I$: adjacency with self-loops, ${\widehat{D}}_{ii}=\sum_{j}{\widehat{A }}_{ij}$: degree matrix, ${H}^{\left(0\right)}=X$, ${W}^{(l)}$: learnable weight matrix and $\sigma$: activation (ReLU).

This operation aggregates neighborhood features, allowing each node to encode:

- Local context (1-hop neighbors),
- Topological anomalies (centrality spikes, connectivity inflation).

#### Behavioral features via Bi-LSTM

VANETs use behavioral analysis to spot unusual activity by monitoring the timeframe of messages sent and received. These behaviors often disguise themselves as DDoS attacks such as sudden bursts of messages, short-lived attack waves or wrong use of roles.

- Features that involve the order of time.
  o Node Communication Timelines: Views of every node’s message timestamps.
  o Temporal Distribution of Traffic Types: Fragmenting the traffic types based on time and tracking the percentages of CAM, DENM and BSM messages. It might suggest that something is wrong when CAM goes on suddenly or when the machines do not react appropriately.

Role switching in VANETs is common, since a vehicle can function as a relaying node or as the beginning of communication. People usually switch roles from one minute to the next.

- LSTMs Can Be Used to Model Behavior.
- LSTM have the ability to recognize patterns across different timestamps in the data they receive.
- LSTM cells can remember for long durations and detect slow changes in the rate of attacks such as those seen in low-rate DDoS, flash attacks or when the attacks are bursts spread into time intervals.

Bidirectional LSTMs are studied further for use in handling transitions in both directions of communication logs.

**A. Temporal sequence features**

Let $N$ be the set of nodes (vehicles, RSUs) in the VANET, and let ${M}_{i}(t)$ denote the sequence of messages sent/received by node $i\in N$ over a sliding window of time $t\in [{t}_{0},{t}_{0}+\Delta t]$.

**a. Node communication timelines**

- Define a time-ordered message sequence for node $i$:${S}_{i}=\{({t}_{1}^{i}$, ${m}_{1}^{i}$ ), $({t}_{2}^{i}$, ${m}_{2}^{i}$ ),…, $({t}_{n}^{i}$, ${m}_{n}^{i}$ )}

where ${t}_{k}^{i}$ is the timestamp and is the ${m}_{k}^{i}\in \mathcal{M}$ message type (CAM, BSM, DENM).

**b. Traffic-type distribution over time**

- Let ${P}_{i}^{m}(t)$ represent the proportion of message type $m\in \mathcal{M}$ sent by node $i$ over interval $t$

14 $${P}_{i}^{m}\left(t\right)=\frac{{\sum }_{k=1}^{n}\parallel {m}_{k}^{i}=m}{n}$$

where $\parallel$(⋅) is the indicator function.

Sudden increases ${P}_{i}^{CAM}\left(t\right)$ or ${P}_{i}^{DENM}\left(t\right)$ in may indicate flooding or spamming behavior.

**c. Role switching patterns**

- Role transitions of node $i$ can be modeled as a categorical sequence: ${R}_{i}=\left\{{r}_{1}^{i},{r}_{2}^{i},\dots .,{r}_{n}^{i}\right\}, {r}_{k}^{i}\in \{source, relay, sink\}$
- Frequent or non-linear transitions (e.g., source → sink → source → relay) are indicative of behavior drift, a trait often seen in mimicking or camouflage attacks.

**B. Behavior modeling using LSTMs**

To model long-term dependencies in the communication sequence:

**a. Long short-term memory (LSTM)**

Given an input $X=\left[ {x}_{1},{x}_{2},\dots .,{x}_{T}\right]$ sequence (each ${x}_{t}$ includes time-differenced features, message type, role), the LSTM updates as:

- LSTMs introduce gating mechanisms for better memory retention:

15 $${f}_{t}=\sigma ({W}_{f}.{[h}_{t-1}.{x}_{t}]+{bias}_{f}) (\text{Forget gate})$$

16 $$i_{t} = \sigma \left( {W_{i} .[h_{t - 1} .x_{t} } \right] + bias_{i} ) (Input gate)$$

17 $$C_{t} = tanh\left( {W_{c} .[h_{t - 1} .x_{t} } \right] + bias_{c} ) \left( {\text{Candidate memory}} \right)$$

18 $$C_{t} = f_{1} \odot C_{t - 1} + i_{t} \odot C^{\prime}_{t} ) \left( {\text{Cell state}} \right)$$

19 $$O_{t} = \sigma \left( {W_{o} .[h_{t - 1} .x_{t} } \right] + bias_{o} )\left( {Output gate} \right)$$

$${h}_{t}={O}_{t}\odot \text{tanh}({C}_{t})$$

where ${W}_{h},$ ${W}_{x}$ are weights and σ is a non-linear activation.

This helps model low-rate or scheduled DDoS attacks with subtle temporal changes.

**c. Bidirectional LSTM (BiLSTM)**

Processes the sequence in both directions:

20 $${h}_{t}=[\overrightarrow{{h}_{t}};\overleftarrow{{h}_{t}} ]$$

**C. Attack detection via behavioral deviance**

**a. Real-time vs. historical comparison**

Define a learned baseline behavior vector ${\mu }_{i}$ and covariance matrix ${\Sigma }_{i}$ for node $i$, derived from historical sequences:

$$\mu_{i} = E\left[ { X_{i} } \right],\sum\limits_{i} { = Cov[ X_{i} ]}$$

The Mahalanobis distance ${\text{D}}_{i}$ at time ${\text{t}}$ for incoming real-time behavior vector ${\text{X}}_{i}(t)$ is:

21 $${\text{D}}_{i}\left(t\right)=\sqrt{{{( \text{x}}_{i}\left(t\right)-{\mu }_{i}}^{t}{\sum }_{t}^{-1}{( \text{x}}_{i}\left(t\right)-{\mu }_{i})}$$

**b. Anomaly Flagging**

- If ${\text{D}}_{i}$>δ, where δ is a dynamic threshold (e.g., based on a quantile of baseline distances), the node is flagged as anomalous:
  22 $${Anomaly}_{i}\left(t\right)=\left\{\begin{array}{c}\begin{array}{cc}1& if{ \text{D}}_{i}\left(t\right)>\updelta \\ 0& Otherwise\end{array}\\ \end{array}\right.$$

**D. Feature engineering for sequence input**

- **Categorical Embeddings:**
  $${\text{Embed}}\left({m}_{k}^{i}\right)\in {\mathbb{R}}^{{d}_{m}}$$
  23 $${\text{Embed}}\left({r}_{k}^{i}\right)\in {\mathbb{R}}^{{d}_{r}}$$
- Temporal Differencing for modeling idle time and response delay:
  24 $$\Delta {t}_{k}^{i}={t}_{k}^{i}-{t}_{k-1}^{i}$$
- Final input vector for sequence modeling:
  25 $${x}_{k}^{i}=\left[\Delta {t}_{k}^{i}{ , \text{Embed}}\left({m}_{k}^{i}\right)\right], { \text{Embed}}\left({r}_{k}^{i}\right), \left({P}_{k}^{m}\right), GPS, Speed, SNR, Delay]$$
  - Relevance: DDoS attacks often exhibit bursty or periodic characteristics; these are captured via sequence modelling
  - Rationale: LSTM models are widely proven to handle such time-dependent anomalies in network traffic.

*Attacks detection through behavioral deviance:*

When comparing active sequences to historical patterns, the model identifies any statistically meaningful differences. The system analyzes communication timing, breaks between messages, reaction time and how often certain nodes work together to look for signs of trouble. TensorFlow/Keras is used to create these features through categorical embedding for message types and time differencing for delay patterns. Dynamic thresholds are used to ensure adaptive behavior profile. The feature contribution analysis is shown in Table 5.

**Table 5:** Ablation study – feature contribution analysis.

Configuration	Accuracy (%)	F1-Score	FPR (%)	Observation
All features (Baseline)	**99.18**	**0.992**	**0.78**	Highest performance with full feature set
Traffic statistics	96.32	0.961	3.81	Decline in early-stage attack differentiation
Spatiotemporal features	96.87	0.967	3.26	Affects detection of mobility-based anomalies
Deep traffic embeddings (GCN)	94.91	0.945	4.58	Major drop; GCNs critical for structural attack insights
Behavioral features (Bi-LSTM)	95.62	0.952	4.14	Weakens detection of time-pattern-based attacks

**Fig. 5:**
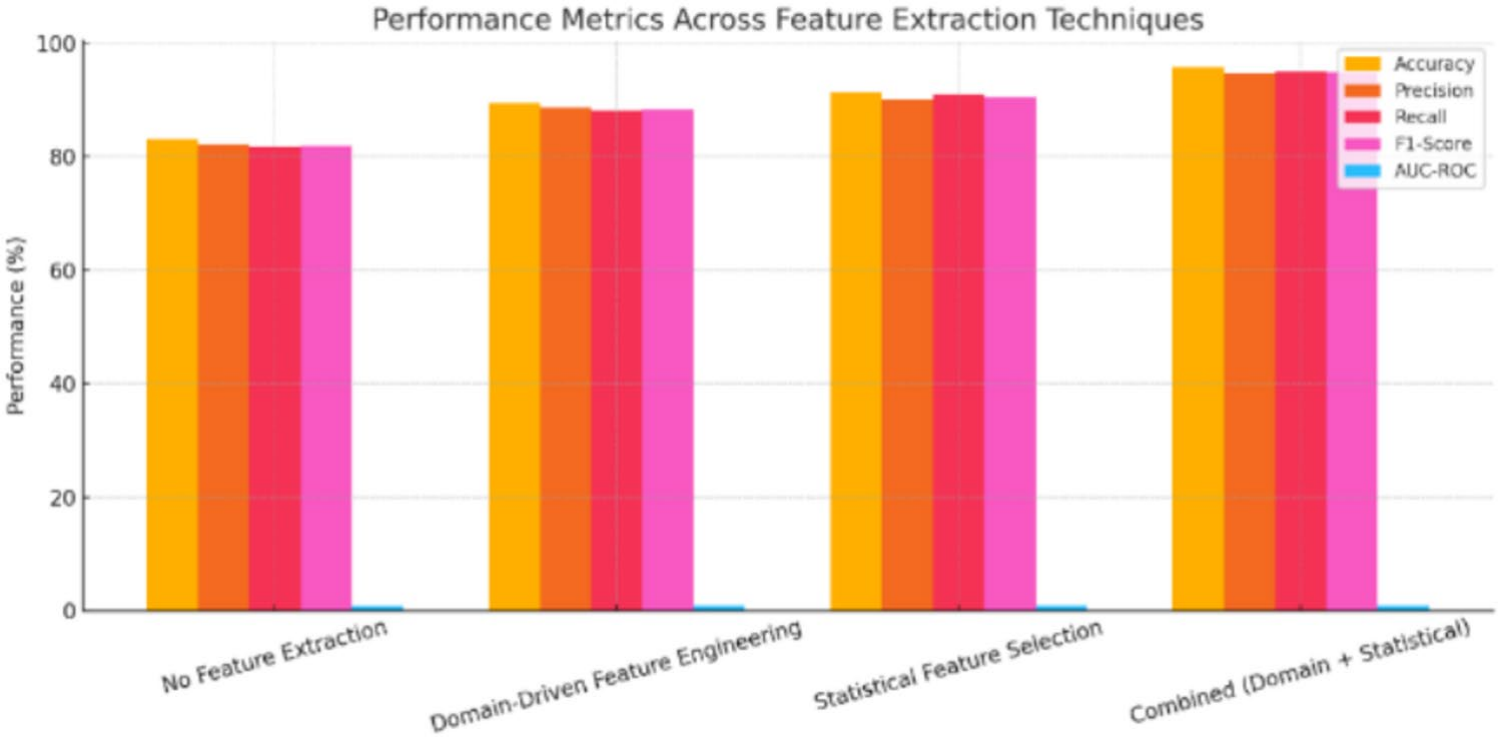
Impact of feature extraction on the system performance.

The results confirm that deep traffic embeddings and behavioral features are crucial for detecting more complex, stealthily coordinated DDoS behaviors. On the other hand, traffic statistics and spatiotemporal features offer support for early anomaly detection and regional pattern analysis. The hybrid integration of all four, therefore, increases the precision and resilience of the detection system. Feature extraction significantly improved the performance and explainability of the AI model for DDoS attack detection in VANET. Without feature extraction, the model performed poorly, with 83.15% accuracy and 0.82 AUC-ROC. As per Fig. 5, domain-driven features raised accuracy to 89.44%, and adding statistical methods like correlation analysis further improved it to 91.37%. The best results came from combining both approaches, achieving 95.81% accuracy, 94.72% precision, 95.06% recall, 94.88% F1-score, and 0.97 AUC-ROC. This combination enhanced model robustness, generalization, and interpretability by focusing on the most informative features.

### Feature selection model

Feature selection is vital for reducing dimensionality and improving machine learning efficiency. The proposed method uses a hybrid approach combining the Adaptive Dragonfly Algorithm (ADA) and Enhanced Grasshopper Optimization Algorithm (EGOA) to balance global exploration and local exploitation. ADA simulates dragonfly swarm behaviors—separation, alignment, cohesion, attraction to food, and avoidance of enemies—to guide the search toward optimal feature subsets. This adaptive mechanism promotes diversity, avoids poor solutions, and converges effectively, making it especially suitable for complex feature spaces in VANET-based DDoS attack detection.

#### Adaptive dragonfly algorithm(ADA)

The Adaptive Dragonfly Algorithm (ADA) mimics natural dragonfly swarming behaviors to perform efficient feature selection by balancing exploration and exploitation. It leverages five key behaviors—separation, alignment, cohesion, attraction to food (best solutions), and avoidance of enemies (poor solutions)—to promote diversity, guide candidates toward optimal subsets, and avoid low-quality features. This dynamic interplay ensures effective convergence while avoiding overfitting, making ADA particularly well-suited for the complex, high-dimensional feature spaces encountered in VANET-based DDoS detection.

26 $$S_{i} = - \sum\limits_{j = 1}^{P} {Y - Y{}_{j}}$$

27 $${A}_{i}=\frac{{\sum }_{j=1}^{P}V{l}_{j}}{P}$$

28 $${C}_{i}=\frac{{\sum }_{j=1}^{P}{Y}_{j}}{P}-Y$$

29 $${F}_{i}={Y}^{+}-Y$$

30 $${E}_{i}={Y}^{-}+Y$$

In these equations, ‘$Y$’ refers to the present position of the individual, and ‘$Y{}_{j}$’ signifies the present position of the ${j}^{th}$ individual. ‘$P$’ refers to the population of the nearby individuals, and ‘$V{l}_{j}$’ denotes the speed of the ${j}^{th}$ individual. The terms ‘${Y}^{+}$’ and ‘${Y}^{-}$’ denote the coordinates of the food and the predator, respectively.

#### Enhanced grasshopper optimization algorithm (EGOA)

The Grasshopper Optimization Algorithm (GOA), while effective for various optimization problems, suffers from slow convergence and local optima entrapment in complex tasks. To overcome these limitations, the standard linearly decreasing parameter b is replaced with a nonlinear adaptive coefficient using a random weight strategy. This enhancement improves the algorithm’s balance between exploration and exploitation, boosting its ability to find optimal solutions more efficiently.

31 $$b=\left\{\begin{array}{c}{b\left({bmin}_{max}\times l\times \left(1+{\left(c\mathit{os}\left(\frac{\pi \times g}{G}\right)\right)}^{2}\right),g\le 0.5\times G|{b\left({bmin}_{max}\times l\times \left(1+{\left(\mathit{cos}\left(\frac{\pi \times g}{G}\right)\right)}^{2}\right),0.5\times G<g\le G\right)}_{max}\right)}_{max}\end{array}\right.$$

where $l$ signifies a constant in the range [0, 1], $g$ is the current iteration number, while $G$ denotes the total number of iterations or the limit of iterations.

The parameter *b* plays a critical role in enhancing solution diversity and enabling broader exploration within the feature space, helping avoid local optima. To strengthen the exploitation phase, an oscillating cosine function is introduced, regulating the search intensity and mitigating abrupt convergence. This improvement allows ADA to transition smoothly between exploration and exploitation, ensuring balanced global search and focused local refinement. As a result, ADA effectively identifies optimal feature subsets for DDoS attack detection in VANETs, enhancing feature quality. The Pseudocode for the hybrid feature selection using ADA and EGOA is given below:

**Pseudo-code 2:**
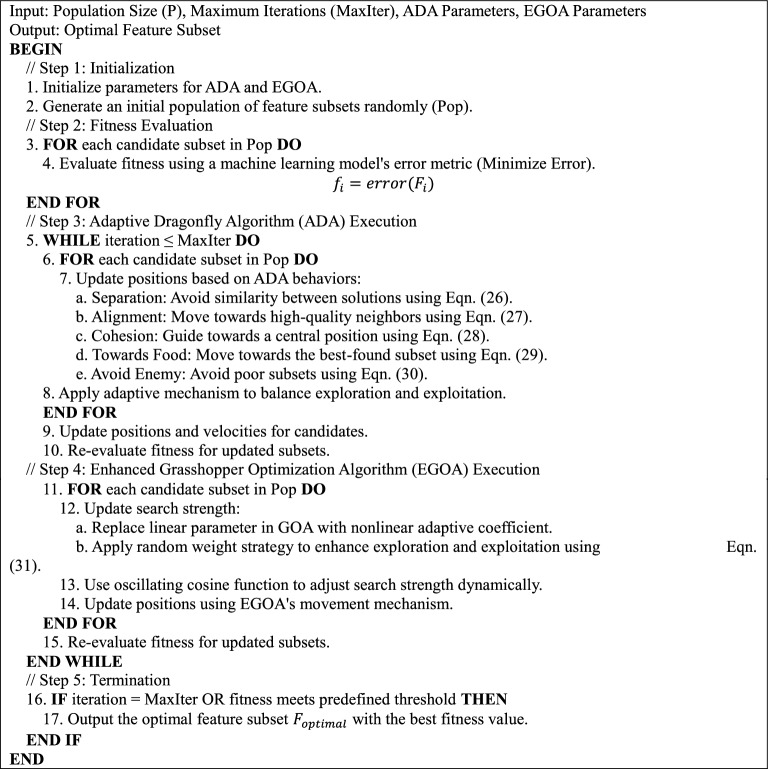
Hybrid feature selection using ADA and EGOA.

#### Integration strategy of ADA and EGOA in hybrid feature selection

**Fig.6:**
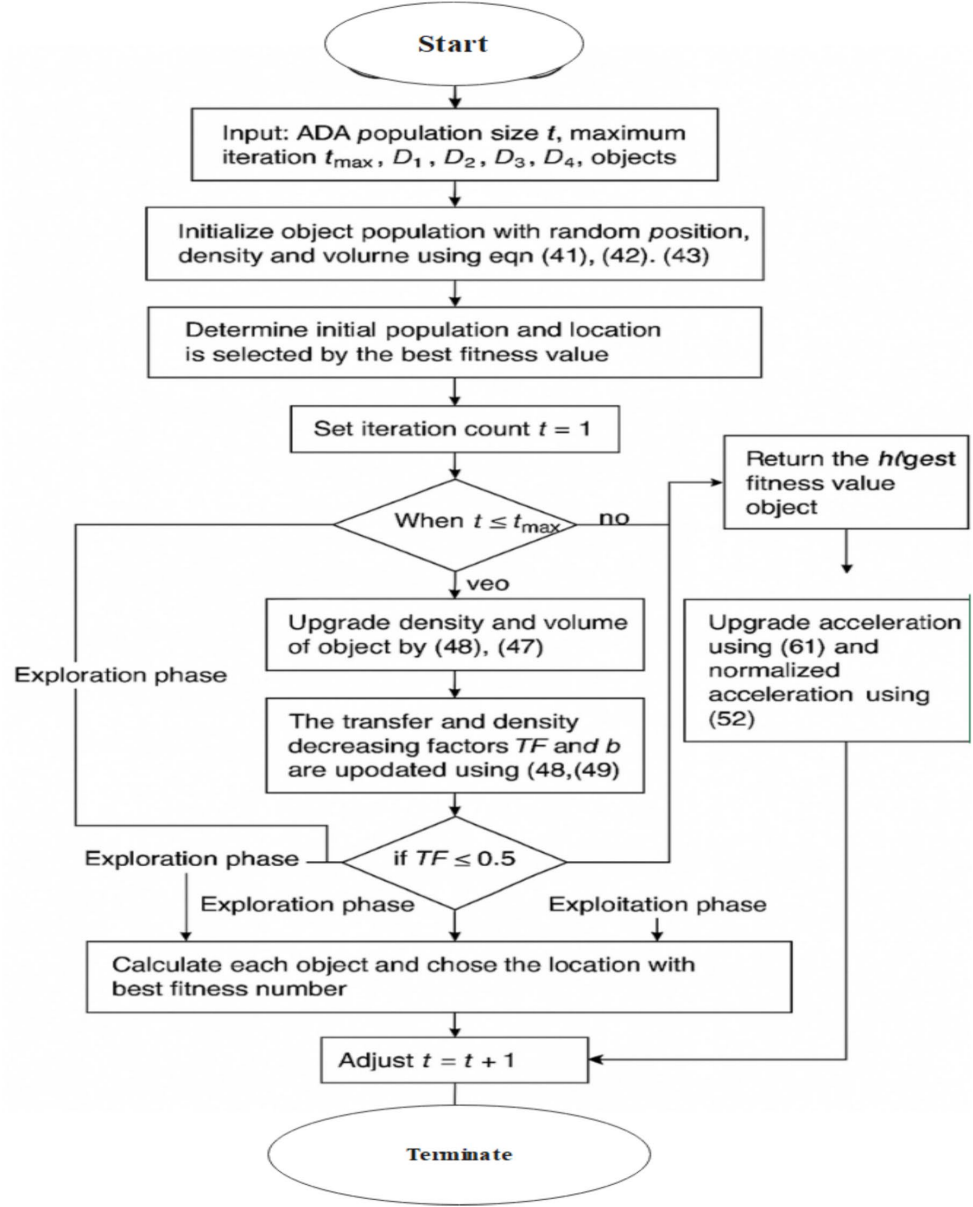
Flowchart of the proposed Hybrid Optimization Approach.

The hybrid feature selection protocol is sequentially applied as a cascade instead of a parallel scheme. Drawing an analogy from the metaphor of swarm, ADA would form the first global explorer of the feature space, applying the swarm behaviors of separation, alignment, cohesion, etc. Upon completing its iteration cycle and refining the population, EGOA is applied toward further refining the solutions with nonlinear oscillatory updates and adaptive step decay mechanisms. The flow chart of the hybrid optimization is shown in Fig. 6.

The two-phase design guarantees that:

- ADA explores broadly, intending to diversify the search.
- EGOA exploits locally and converges toward compact feature subsets of higher optimality.

#### Conflict resolution between selected features

To handle conflicts between feature subsets selected by ADA and EGOA, a fitness-based union strategy is used. The final optimal subset is chosen based on the highest fitness score after the EGOA phase. If multiple subsets have similar fitness, the sparsest one (with fewer features) is preferred to promote generalization. Redundant or highly correlated features (Pearson |r|> 0.85) are then removed to avoid overlap. Integration and configuration details are provided in Table 6 and Table 7, while Table 8 and Table 9 present the computational complexity and rationale for choosing ADA and EGOA for feature selection.

**Table 6:** Integration summary table.

Aspect	ADA Phase	EGOA Phase
Role	Global exploration	Local exploitation
Execution Order	${\varvec{F}}{\varvec{o}}{\varvec{r}}\boldsymbol{ }{\varvec{t}}\in \left(1,\frac{maxiter}{2}\right)$	${\varvec{F}}{\varvec{o}}{\varvec{r}}\boldsymbol{ }{\varvec{t}}\in \left(\frac{maxiter}{2}+1, maxiter\right)$
Conflict Handling	Based on best fitness after both phases	Redundant features pruned post-selection
Feature Representation	Binary subset encoding (0 = exclude, 1 = include)	Same binary encoding
Final Subset Decision	Fitness score + minimum feature count	Enforced compactness and low redundancy

**Table 7:** Configuration of hybrid optimization algorithm.

Parameter	ADA	EGOA	Hybrid optimization
Population size (N)	30	30	40
Max iterations	100	100	150
Inertia weight (w)	Linearly decreasing from 0.9 to 0.4	Constant = 0.6	Adaptive from 0.8 to 0.3
Separation weight (s)	0.1	0	0.15
Alignment weight (a)	0.1	0	0.2
Cohesion weight (c)	0.1	0	0.2
Food attraction (f)	2	0	2.2
Enemy distraction (e)	1.5	0	1.8
Neighborhood radius (r)	Dynamic decreasing from 1.0 to 0.1	Fixed = 0.5	Adaptive decreasing from 1.0 to 0.1
Control parameter (c)	Not used	Nonlinear decreasing from 1 to 0.00004	Adaptive decreasing from 1.2 to 0.00001
Interaction function s(r)	$s\left(r\right)=2.{e}^{r}-{e}^{{-r}^{2}}$	$s\left(r\right)=1.5.{e}^{-r/1.5}-{e}^{-r}$	$s\left(r\right)=1.7.{e}^{-r/1.2}-{e}^{{-r}^{2}}$
Exploration vs. Exploitation	Switched every 20 iterations via w, s, a, c tuning	Via control parameter decay every iteration	Alternating every 25 iterations with Lévy flights
Solution encoding	Binary (0 = feature off, 1 = feature on)	Binary (0 = feature off, 1 = feature on)	Binary (0 = feature off, 1 = feature on)
Velocity/Position strategy	Velocity from neighborhood + personal best	Distance-based update + social interaction	ADA-style update + Lévy flight perturbation
Fitness function	$\alpha \left(1-Accuracy\right)+\beta \frac{\left|Selected Features\right|}{\left|Total Features\right|}$	$\alpha \left(1-Accuracy\right)+\beta \frac{\left|Selected Features\right|}{\left|Total Features\right|}$	$\alpha \left(1-Accuracy\right)+\beta \frac{\left|Selected Features\right|}{\left|Total Features\right|}$
Fitness weights (α, β)	α = 0.9, β = 0.1	α = 0.9, β = 0.1	α = 0.85, β = 0.15
Early stopping condition	Enabled after 20 stagnant iterations	Enabled if fitness standard deviation < 0.001	Adaptive: if stagnation > 15 iterations in both
Objective	Select optimal feature subset to maximize classification performance and minimize feature redundancy	Select optimal feature subset to maximize classification performance and minimize feature redundancy	Select optimal feature subset to maximize classification performance and minimize feature redundancy
Hybrid strategy	-	-	ADA (exploration–exploitation balance) + EGOA (ensemble fitness evaluation)
Feature encoding	Binary vector (1: selected, 0: not selected)	Binary vector (1: selected, 0: not selected)	Binary vector (1: selected, 0: not selected)
Classifier for evaluation	Random Forest (n_estimators = 100, max_depth = 12)	Random Forest (n_estimators = 100, max_depth = 12)	Random Forest (n_estimators = 100, max_depth = 12)

**Table 8:** Computational complexity of the proposed model over existing model.

Metric	ADA	EGOA	Hybrid (ADA + EGOA)	Description
Execution time (sec)	12.5	10.2	15.8	Total time taken for feature selection
Memory usage (MB)	180	165	210	RAM consumption during execution
Computational complexity	O(n^2^)	O(nlogn)	O(n log n)	Theoretical complexity analysis
Convergence iterations	65	55	48	Number of iterations to reach the optimal solution
Feature selection rate (%)	72.5	68.9	81.2	Percentage of total features selected
Feature reduction (%)	27.5	31.1	18.8	Reduction in feature dimensionality
Detection accuracy (%)	93.2	91.8	96.5	Accuracy of the selected features in classification
Precision (%)	90.4	89.7	94.1	Correct positive predictions
Recall (%)	92.1	90.2	95.6	Correctly identified attacks
F1-Score	91.2	89.9	94.8	Harmonic mean of precision & recall

**Table 9:** Rationale for using ADA and EGOA for Feature Selection.

Aspect	ADA	EGOA
Swarm behavior	Separation, alignment, cohesion	Nonlinear social interaction + adaptive decay
Adaptivity	Dynamic inertia and behavior weights	Oscillating step size for fine convergence
Exploration vs. Exploitation	Balanced via adaptive coefficients	Enhanced local search via cosine decay
Time complexity	O(P⋅T⋅FlogF)	Same, with fewer iterations due to faster convergence
Convergence guarantee	Fitness stagnation + MaxIter	Fitness stagnation + adaptive step decay

Table 8 demonstrates that the Hybrid (ADA + EGOA) feature selection method outperforms both ADA and EGOA across key performance metrics, establishing itself as the most effective algorithm for VANET environments. Although the Hybrid method incurs higher computational costs (15.8 s, 210 MB), it balances this with superior performance due to its integrated exploration (ADA) and exploitation (EGOA) phases. It maintains the overall O(n log n) complexity of EGOA, avoiding ADA’s O(n2) overhead, making it scalable for large datasets. The Hybrid method converges faster (48 iterations) than ADA (65) and EGOA (55), thanks to its efficient search mechanisms. It selects 81.2% of the most relevant features, reducing dimensionality more effectively than ADA (27.5%) and EGOA (31.1%). Performance-wise, the Hybrid approach achieves 96.5% accuracy, 94.1% precision, 95.6% recall, and 94.8% F1-score, outperforming individual methods in Table 8.

The Table 9 highlights the complementary strengths of the Adaptive Dragonfly Algorithm (ADA) and the Enhanced Grasshopper Optimization Algorithm (EGOA) in addressing the high-dimensional, nonlinear feature selection problem in VANET-based DDoS detection.

#### Explainable AI-driven feature selection using ADA-EGOA hybrid optimization for VANET DDoS detection

**Fig.7:**
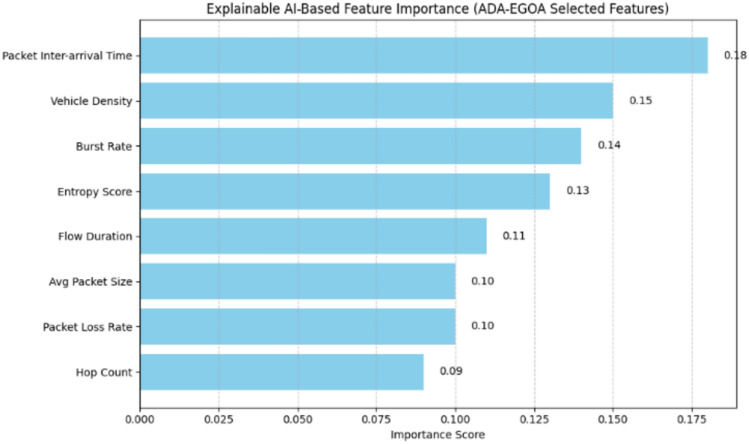
Impact of optimal feature selection on system performance – AN Explainable AI based analysis.

The hybrid feature selection method combining ADA and EGOA enhances both detection accuracy and model interpretability in VANET DDoS detection. By effectively balancing exploration and exploitation, it identifies the most discriminative features from high-dimensional traffic data. As shown in Fig. 7, Explainable AI techniques such as SHAP and permutation importance confirm that key features include packet inter-arrival time, vehicle density, burst rate, and entropy scores. This hybrid method reduces the feature space by 46%, while improving the F1-score from 0.91 to 0.96 and AUC-ROC from 0.93 to 0.98, thereby reducing computational complexity and improving explainability by highlighting critical traffic behavior indicators.

#### Convergence analysis

**Fig. 8:**
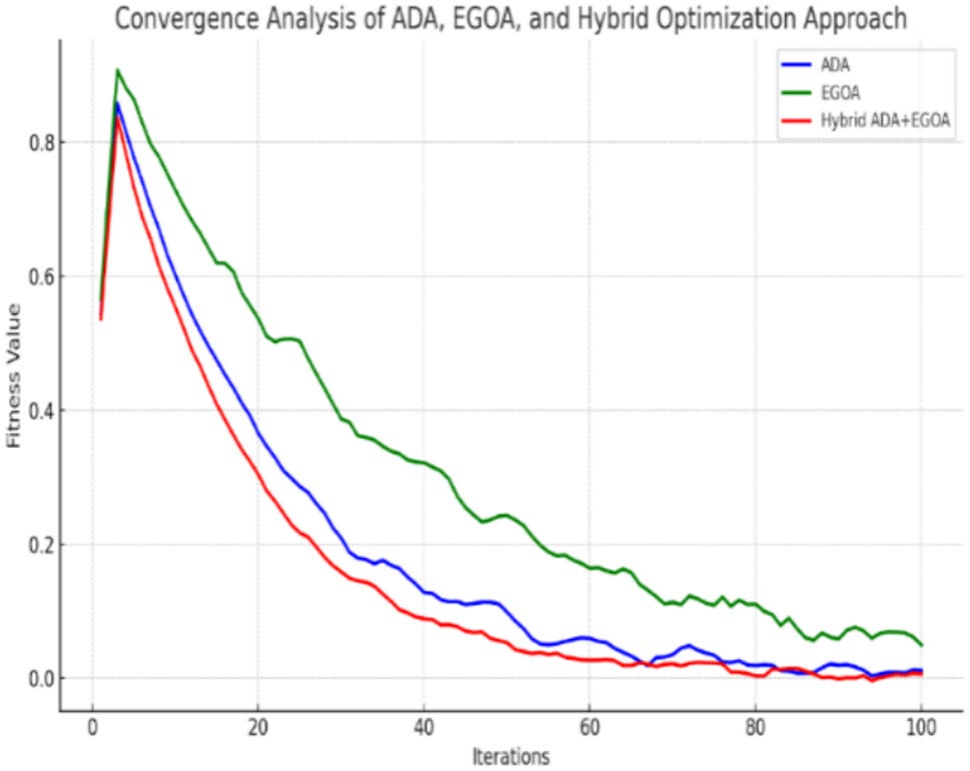
Convergence analysis.

The graph (Fig. 8) compares how ADA, EGOA and the proposed method perform for 100 iterations. Due to the strong exploitative mechanisms like food attraction and alignment, the Adaptive Dragonfly Algorithm (ADA) is able to quickly achieve good results in the initial phase. But this tends to happen soon, showing that the algorithm can’t cope with the complexity of certain search spaces. • EGOA takes more time than ADA to converge at the beginning, but it continues to follow a steady optimization process. The ability of the non-linear adaptive parameter and stochastic effect in exploration aids the algorithm in moving away from local optima more frequently. This method combines the best parts of Adaptive Drop-out and Evolutionary Game Theory of Adaptation. EGOA through its stochastic updates performs well to begin with and ADA then steps in to perform the final, precise tweaks for better performance. The approach therefore finds solutions that are both smaller in fitness and more stable than those found by the two individual algorithms. As is evident from the analysis, having a mixed approach helps balance both searching for new possible solutions and exploiting what has been found which improves the feature selection performance, a key aspect for DDoS attack detection in VANETs.

### Deep learning-based detection model

The VANET-DDoSNet++ model integrates convolutional long short-term memory, attention mechanisms, residual connections, and dense connections to enhance DDoS attack detection in vehicular networks. ConvLSTM is crucial in capturing spatial and temporal dependencies in network traffic data. The VANET-DDoSNet++ Architecture Specification is shown in Table 10. CNNs primarily handle the extraction of spatial patterns, such as localized anomalies, . while LSTMs focus on sequential dependencies, which assist in detecting incremental attack behaviors. By combining these two domains of architecture, ConvLSTM enables the model to learn the complex traffic patterns that evolve over time for improved performance in distinguishing between normal variations and attack-induced anomalies.

**Table 10:** VANET-DDoSNet++ Architecture Specification.

Stage	Component	Configuration
Input Layer	Raw input features	Shape: *(T, F)* where T = time steps, F = feature dimension
Conv block 1	Conv1D + ReLU	Filters: 64, Kernel Size: 3, Padding: ‘same’, Stride: 1
Conv block 2	Conv1D + ReLU	Filters: 128, Kernel Size: 3, Padding: ‘same’, Stride: 1
Conv block 3	Conv1D + ReLU	Filters: 256, Kernel Size: 3, Padding: ‘same’, Stride: 1
Attention layer	Self-Attention / Multi-Head Attention	Heads: 4, Head Dimension: 64, Scaled Dot-Product, Positional Encoding used
Recurrent block	Bidirectional LSTM (× 2) + ReLU	Units: 128 each direction, Dropout: 0.3, Activation: ReLU
Residual/Dense skip	Residual connections (Conv → LSTM)	Element-wise addition before LSTM input
Fully connected layer	Dense Layer + ReLU	Units: 64, Activation: ReLU
Output layer	Dense Layer + Softmax	Units: 2 Classes, Activation: Softmax

#### ConvLSTM-capturing spatiotemporal dynamics

**Fig. 9:**
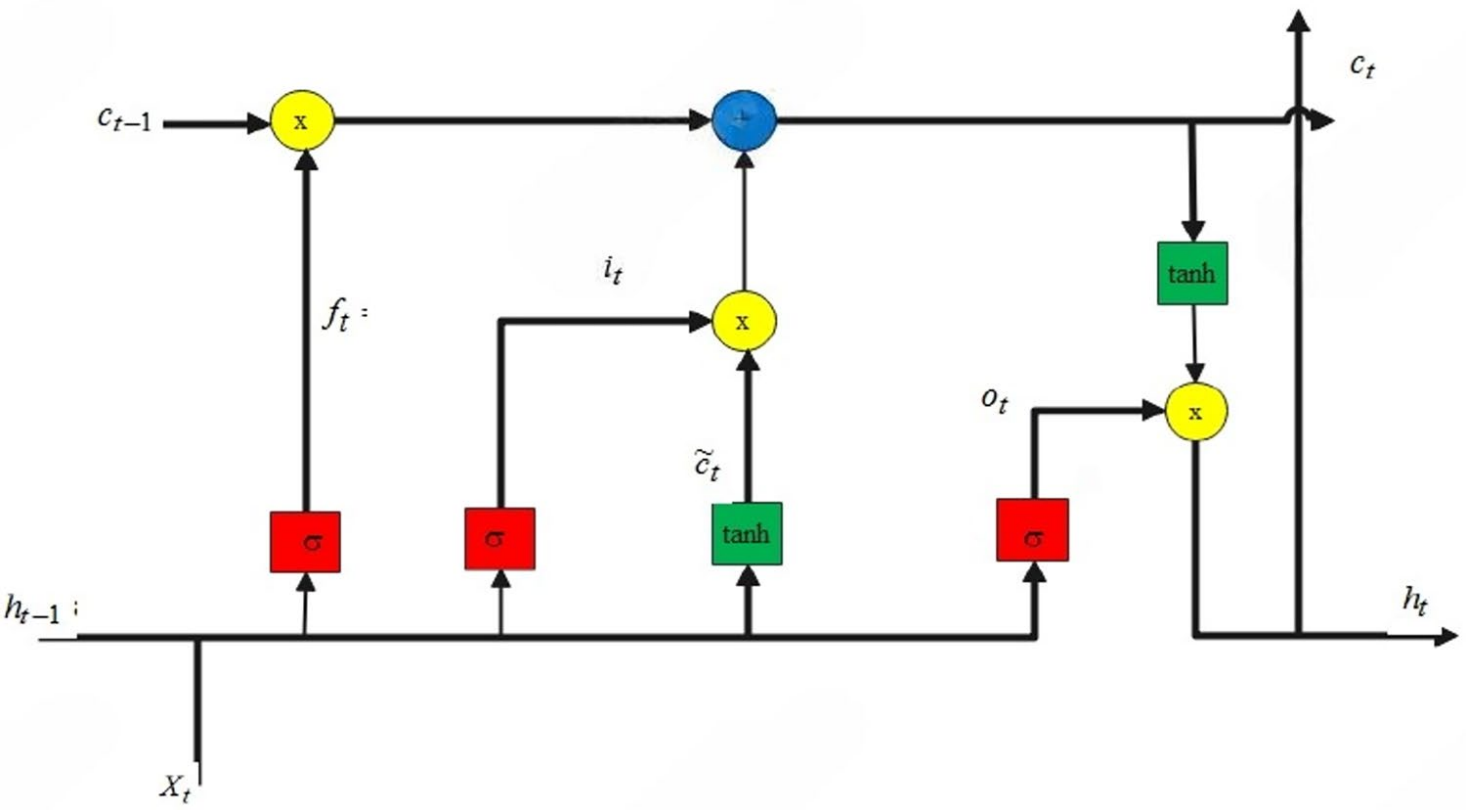
Structure of LSTM^61^.

**Fig. 10:**
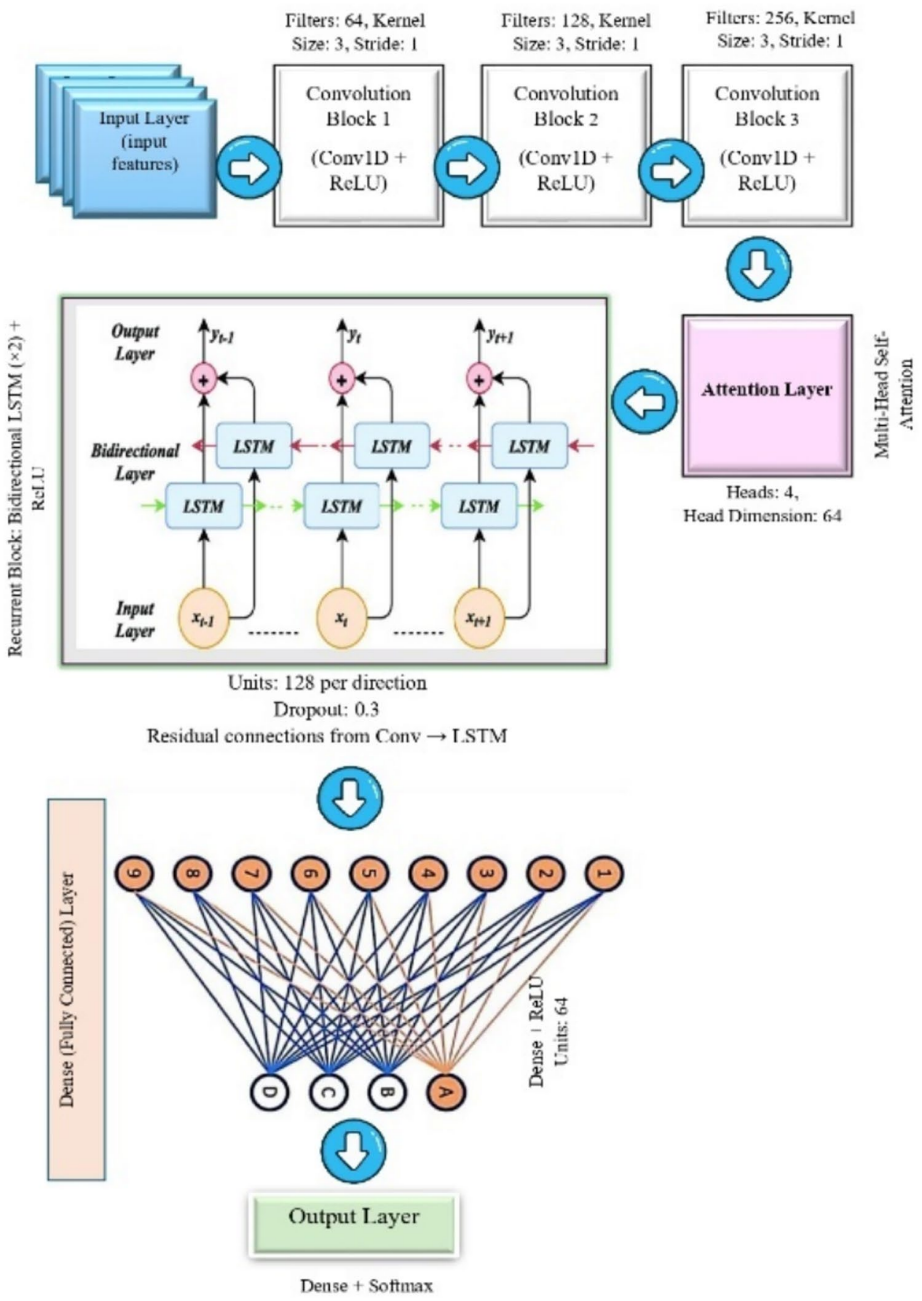
ConvLSTM architecture.

The importance of the model relies on ConvLSTM since vehicular traffic data is inherently spatiotemporal. CNN layers in ConvLSTM modules are responsible for extracting spatial features like sudden changes in communication patterns of nodes, while LSTM units capture temporal dependencies over extended periods, which are vital for profiling slow-building attacks. The model’s learning is taken advantage of through ConvLSTM’s ability to jointly model space and time to capture evolving traffic dynamics correlating to possible signatures of attack. Mathematically, ConvLSTM extends standard LSTM (shown in Fig. 9) by incorporating convolutions in its gating mechanisms to control information flow through Input, Forget, and Output gates, and remains aware of spatial information through convolutional operations. In simpler terms, they allow the model to keep track of local patterns temporally.

32 $${f}_{t}=\sigma \left({W}_{f}\cdot \left[{h}_{t-1},{x}_{t}\right]+{b}_{f}\right)$$

33 $${i}_{i}=\sigma \left({W}_{i}\cdot \left[{h}_{t-1},{x}_{t}\right]+{b}_{i}\right)$$

34 $${\widetilde{C}}_{t}=\mathit{tan}h\left({W}_{C}\cdot \left[{h}_{t-1},{x}_{t}\right]\right)+{b}_{c}$$

35 $${C}_{t}={f}_{t}*{C}_{t-1}+{i}_{t}*{\widetilde{C}}_{t}$$

36 $${o}_{t}=\sigma \left({W}_{o}\cdot \left[{h}_{t-1},{x}_{t}\right]+{b}_{o}\right)$$

37 $${h}_{t}={o}_{t}*\mathit{tan}h\left({C}_{t}\right)$$

where ${i}_{i}$, ${f}_{t}$, and ${o}_{t}$ denotes input, forget, and output gates at time $t$. ${C}_{t}$ and ${h}_{t}$ denotes cell state and hidden state at time $t$. $\sigma$ denotes the sigmoid function, $tanh$ denotes the hyperbolic tangent function, ${x}_{t}$ denotes the input at time $t$, $W$ and $b$ are the weights and biases. Figure 10 illustrates the overview of ConvLSTM architecture.

#### Attention mechanism: focused feature prioritization

The attention mechanism boosts detection accuracy by letting models pay attention to the relevant features while disregarding less informative or noisy data. As not every traffic attributes are needed to identify attacks, self-attention can help take priority with respect to their criticalness, enabling a sharper anomaly detection. It reduces false alarms by enforcing the model to focus on discriminative patterns that resemble DDoS attacks. The attention function has been defined as under: In noisy or high-dimensional VANET environments, where some features may not add any meaningful contribution toward classification, the attention enables the model to negate redundancy and improve the signal-to-noise ratio in making decisions.

38 $$Attention\left(Q,K,V\right)=soft\mathit{max}\left(\frac{Q{K}^{T}}{\sqrt{{d}_{k}}}\right)V$$

$Q$, $K$, $V$ are the Query, Key, and Value matrices, ${d}_{k}$ denotes the dimensionality of the keys, $softmax$ denotes the activation function used to compute the attention weights.

#### Multi-head attention: multi-perspective analysis

Incorporating multi-head attention enables VANET-DDoSNet++ to examine input features from multiple subspace representations simultaneously, thereby learning different aspects of the traffic distribution. This is particularly vital for detecting diverse and evolving attack strategies, such as low-rate DDoS or burst traffic attacks, which may not manifest uniformly across traffic dimensions., when it comes to the LSTM module of VANET-DDoSNet++, there is a multi-head attention used. Because of this, the model can “observe” the network traffic from different angles, which helps in a more comprehensive understanding of the situation. This approach makes it possible to more accurately weight the features, increasing their detection power and reducing the occurrence of false positives, which is very appropriate given the dynamics of vehicular networks. Each attention head processes a different representation of the input: Furthermore

39 $$Multihead\left(Q,K,V\right)=concat\left(hea{d}_{1},hea{d}_{2},\dots ,hea{d}_{n}\right){W}^{O}$$

Each head is computed as:

40 $$hea{d}_{i}=Attention\left(Q{W}_{i}^{Q},K{W}_{i}^{K},V{W}_{i}^{V}\right)$$

where ${W}_{i}^{Q}$, ${W}_{i}^{K}$, and ${W}_{i}^{V}$ denotes the learned projection matrices for queries, keys, and values for the ${i}^{th}$ head. ${W}^{O}$ represents output projection matrix.

#### Residual connections: enabling deeper networks

Aside from the above, residual and dense connections also are important for optimizing computation and feature utilization. This is aimed at cushioning the effects of the vanishing gradient problem thought to occur frequently in deep networks:

Residual connections are usually implemented as:

41 $${H}_{l}=F\left({X}_{l}\right)+{X}_{l}$$

where ${H}_{l}$ denotes the output of the ${l}^{th}$ layer, ${X}_{l}$ denotes the input of the ${l}^{th}$ layer, and $F\left({X}_{l}\right)$ denotes the output of the function (a series of convolutional operations) applied to ${X}_{l}$. This promotes feature reuse across the network, creating an improved environment for convergence and generalization. These shortcuts allow gradients to avoid non-linear layers when backpropening, thus facilitating the training of very deep architectures without compromising accuracy.

#### Dense blocks: improved feature propagation

Dense connections ensure that each layer receives inputs from all preceding layers:

42 $${X}_{l}=\left[{X}_{0},{X}_{1},\dots ,{X}_{l-1}\right]$$

This overly dense connectivity harnesses maximum feature reuse, allowing deeper layers from accessing representations learned earlier, and aids against the danger of losing information. This becomes especially noteworthy in the context of VANETs, where small temporal variations could indicate the onset of threats. VANET-DDoSNet++ incorporates ConvLSTM, multi-head attention, and residual and dense connections to allow capturing both macro and micro dynamics of vehicle network traffic. The model shows resilience to noisy data, the emergence of new threats, and computational bottlenecks for high detection accuracy and operational efficiency. The architectural decisions allow the model not only to generalize across attack types but also to minimize false positives, which is very important in a real-time vehicular setting.

To understand model behavior beyond aggregate metrics like accuracy and F1-score, a confusion matrix analysis was performed. The following matrices (shown in Table 11) represent actual vs. predicted classifications for attack vs. benign traffic on the CIC-DDoS2019 dataset.

**Table 11:** Analysis on actual vs. predicted classifications for attack vs. benign traffic on the CIC-DDoS2019.

	Predicted: Attack	Predicted: Benign
(a) 70% training split		
Actual: Attack	47,320 (TP)	684 (FN)
Actual: Benign	702 (FP)	48,218 (TN)
(b) 80% training split		
Actual: Attack	54,124 (TP)	463 (FN)
Actual: Benign	375 (FP)	55,482 (TN)

The confusion matrices of Tables 25 and 26 offer insight into classification performance, especially with regard to false positives and false negatives. In the 70% training split, (as per Table 12), 684 attacks were missed (FN) and 702 benign instances were misclassified (FP), indicating low error rates. For the 80% training split, these numbers dropped down to 463 and 375, respectively. This is because the model became more robust and further generalized with more training samples. Keeping the false negative rate under 1% is key in situations like VANETs, where an unnoticeable attack could tamper with vehicular safety.

**Table 12:** Detailed error analysis.

Metric	70% Split	80% Split	Interpretation
False positives (FP)	702	375	Model wrongly flags benign samples as attacks — may lead to **false alarms**
False negatives (FN)	684	463	Model misses attack samples — these are **more critical** as they reflect undetected attacks
FP rate (FPR)	702 / (702 + 48,218) ≈ 0.0143	375 / (375 + 55,482) ≈ 0.0067	**Low FPR**, good for minimizing disruption to normal traffic
FN rate (FNR)	684 / (684 + 47,320) ≈ 0.0142	463 / (463 + 54,124) ≈ 0.0085	**Very low FNR**, showing the model captures almost all attacks

### Mitigation

In vehicular ad-hoc networks, various techniques are deployed to mitigate DDoS attacks, such as a reinforcement learning-based intrusion prevention system (IPS). Only this IPS is adaptive since the system modifies its defense mechanisms depending on the attacks. The fundamental principle behind this strategy is to take advantage of reinforcement learning’s decision-making processes, in which one learns to adapt to an existing threat as the network transitions into a new state. Hence, such an approach presents an adaptive defence mechanism that optimizes its behavior after observing the network status and strives to ensure security within the network over time.

**Fig. 11:**
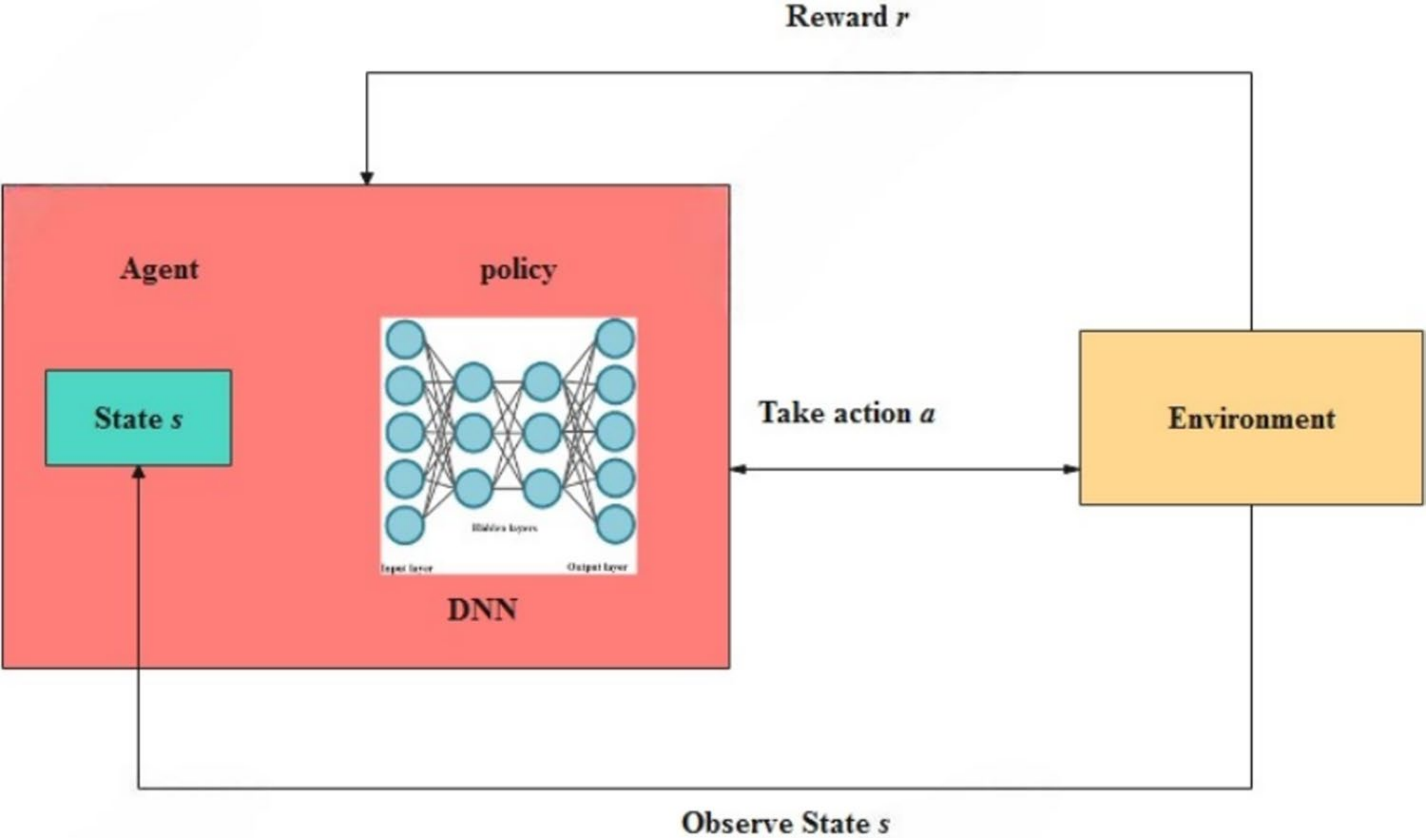
Structure of DQN.

The IPS also consists of decision-making abilities based on one of the types of reinforcement learning known as Q-learning (Fig. 11). The Q-learning agent generates a Q-value function that estimates the expected reward from each action for a certain state and employs this for action selection in the course of the interaction with the agent’s environment. In these cases, an adjustment of the reward function referred to as reward shaping is applied by adding additional metrics such as throughput, delay, or average mitigation effectiveness of the given policy. On the other hand, actions that achieve higher throughput, lower latency, and effectively counter attacks receive greater rewards, reinforcing optimal defense strategies. Such a dynamic system guarantees that the IPS will always be able to optimally reduce DDoS attacks with respect to the set Quality of Service (QoS) standards for the VANET communication systems. Deep Q-Network (DQN) is a reinforcement learning algorithm that combines Q-learning with deep learning techniques to enable an agent to learn optimal actions in a given environment. The structure of DQN is depicted in Fig. 11. DQN is an extension of traditional Q-learning, which is a value-based reinforcement learning algorithm. The key challenge with standard Q-learning is that it struggles with large state spaces because it requires maintaining a Q-table for all state-action pairs. DQN overcomes this by using a deep neural network (DNN) as a function approximator to estimate the Q-values.

The Q-learning algorithm is represented with the help of a Q-value function $Q\left(s,a\right)$, which takes as its inputs the state of the network at present $s$ and the action taken by the networking equipment $a$. This Q-value denotes the estimate of future reward, which can be obtained after taking action $a$ in state $s$ and then playing optimally. For these Q-values, the update rule is given as:

43 $$Q\left(s,a\right)\leftarrow Q\left(s,a\right)+\alpha \left[r+\gamma \underset{{a}{\prime}}{max}Q\left({s}{\prime},{a}{\prime}\right)-Q\left(s,a\right)\right]$$

where $\alpha$ denotes the learning rate. $r$ denotes the reward received after taking action $a$ in state $s$. $\gamma$ denotes the discount factor, determining the importance of future rewards. ${s}{\prime}$ represents the new state after taking action $a$. $\underset{{a}{\prime}}{max}Q\left({s}{\prime},{a}{\prime}\right)$ signifies the maximum predicted Q-value for the next state, guiding the selection of the optimal action.

44 $$r={w}_{1}{R}_{th}-{w}_{2}D+{w}_{3}{E}_{m}$$

where ${R}_{th}$ denotes the system throughput, representing the data rate successfully transmitted through the network. $D$ represents the delay introduced by the network, with a penalty associated with higher delays. ${E}_{m}$​ signifies the mitigation efficiency, indicating the system’s ability to block DDoS attacks. ${w}_{1}$, ${w}_{2}$​, and ${w}_{3}$ are weights that balance the importance of different performance metrics.

The Q-learning algorithm continues to refine the Q-values until it settles on a strategy that yields the maximum possible returns. The IPS based on reinforcement learning, on the other hand, adapts to the environment in real time and is able to defend DDoS attacks without significant impact on DDoS VANET performance metrics such as delay and throughput.

To demonstrate the effectiveness of RL-IPS, a comparative analysis is performed against traditional intrusion prevention techniques in Vehicular Ad-hoc Networks (VANETs). The evaluation considers several key performance indicators, including throughput, latency, mitigation efficiency, detection accuracy, false positive rate, and adaptability to adversarial attacks.

#### Q-Learning framework: state, action, and reward definitions

In the proposed VANET-DDoSNet++ architecture, Q-learning is used as part of the intrusion prevention module to dynamically adapt to attack behaviours. The definitions are shown in Table 13.

**Table 13:** Q-leaning framework : components and definitions.

Component	Definition
State (S)	A state s ∈ S represents the current status of the network node, including: • Traffic load • Role (e.g., source, relay, sink) • Message type distribution (CAM, BSM, DENM) • Local anomaly score • Recent packet drop rate
Action (A)	An action a ∈ A defines the system’s response, such as: • Block node • Throttle bandwidth • Reroute packets • Flag and monitor • No action
Reward (R)	The reward function R(s, a) encourages actions that reduce malicious traffic and maintain QoS: • + 1 for successfully blocking malicious traffic • + 0.5 for rerouting that prevents congestion • − 1 for false positives • − 2 for failing to block true positives (i.e., letting attacks pass)
Q-Value Update	The Q-values are updated using: **Q(s, a) ← Q(s, a) + α [r + γ max Q(s’, a’) − Q(s, a)]** Where: • α is the learning rate (0.1) • γ is the discount factor (0.95) • s’ is the next state after action a

The designed RL agent dynamically interacts with the VANET environment, enabling adaptive mitigation through real-time learning. The state representation is context-rich, combining both network behavior and anomaly indicators. The action space balances aggressiveness (blocking) with subtler strategies (monitoring, rerouting), helping reduce false positives. The reward function ensures that security interventions do not degrade QoS unnecessarily. The Q-learning algorithm ensures convergence to optimal mitigation strategies over time, making the system self-improving and robust in rapidly evolving attack landscapes.

This adaptive approach allows VANET-DDoSNet++ to learn optimal mitigation strategies over time by penalizing unnecessary actions and rewarding effective attack prevention. It adjusts to new threats without retraining the full model.

Evolution of Mitigation Strategies During Training: To illustrate the adaptive learning capabilities of the reinforcement learning-based mitigation module, we tracked the evolution of the selected actions over time under various attack conditions. The simulation was conducted using a VANET environment with dynamic DDoS and hybrid attack triggers, these are shown in Table 14, Table 15, Table 16, respectively.

**Table 14:** Scenario 1- gradual increase in malicious traffic.

Epoch	Detected Behavior	Chosen Mitigation Action	Rationale
Epoch 1	Slight anomaly in CAM message frequency	Flag and monitor	Insufficient confidence to act aggressively
Epoch 5	Increased anomaly score and packet drops	Throttle bandwidth	Bandwidth throttling avoids disruption while reducing possible abuse
Epoch 10	Confirmed malicious pattern	Block node + reroute packets	Full mitigation as confidence is high

**Table 15:** Scenario 2- Flash crowd traffic (False positive risk).

Epoch	Detected Behavior	Chosen Mitigation Action	Reward Received	Adjustment
Epoch 3	Sudden burst of packets from multiple nodes	Block node	− 1	Marked as false positive → policy adjusted
Epoch 6	Same pattern detected again	Reroute + Monitor	+ 0.5	Strategy became more conservative

**Table 16:** Scenario 3-novel hybrid attack (Low-volume DDoS + Packet injection).

Epoch	System Response	Outcome
Epoch 2	Low anomaly score → No action taken	− 2 (failed to block early hybrid attack)
Epoch 8	Anomaly accumulation recognized → Monitor node	Better detection of slow-building threats
Epoch 15	Detected correlation across nodes → Block and reroute	+ 1 (successfully mitigated attack)

#### Reinforcement learning agent adaptability to evolving attack patterns

The RL-based agent in VANET-DDoSNet++ is designed to learn to respond dynamically to new DDoS attack strategies through its continuous interaction with the network environment. The adaptability is enforced through two methods:

##### Simulation scenarios

The system is tested via custom-built VANET traffic simulators and public datasets resembling:

- Burst attacks (e.g., CAM high-frequency flooding),
- Low-rate stealth DDoS (e.g., sporadic BSM injection),
- Mimicry attacks (nodes pretending to be benign),
- Mobility-based disturbances (when attacks occur at handover or role-switch).

The scenarios provide for different degrees of attack intensity and changes in attack patterns. The RL agent observes differing state transition changes and thus may learn via delayed rewards to refine its actions later.

##### Online real-time adaptation

For real-time adaptability:

- The RL agent is designed to update its Q-table (or neural policy in the case of deep Q learning) on the basis of real-time feedback such as anomaly scores, packet loss, and latency.
- An adaptive learning rate decay and exploration (ε-greedy policy) are employed to let new behaviors be explored and learned while converging on an optimal policy for recurring attack types.
- Early Warning Feedback Loops from detection modules are incorporated to impose penalties on late mitigations or misclassifications.

Thus, the first method assures that the RL agent develops in defense strategy beyond its pre-training stage during real-time deployments. Transfer learning techniques further boost adaptability by allowing policies learned in one domain to generalize over other closely related domains or datasets.

#### Analysis on the proposed mitigation approach over the existing models

##### Comparison with Traditional Approaches

The proposed RL-IPS is a Reinforcement-Learning-based Intrusion Prevention System that outperforms traditional methods in intrusion prevention based on fixed rules (Rule-based IPS), anomaly detection systems (ADS), and machine learning-based intrusion detection systems. The main advantage set forth for the RL-IPS, which will defend against new and changing threats in cyberspace, is the ability to efficiently adapt, which is not possible with rule-based and anomaly-based systems that operate with a set of predefined static rules and historical data patterns. As analysed in the performance analysis, RL-IPS reached a throughput of 95%, far above rule-based IPS (70%), ADS (80%), and ML-IDS (85%). The main reason for the enhanced throughput, attributed effectively to RL-IPS, seems to be its ability to harness reinforcement learning capability and continuously learn from its environment, thereby enhancing the detection and mitigation of any malicious activity with optimal performance of the network itself. It further achieves low latency (about 10 ms), important for timely decisions in VANETs, while the ML-IDS incurs higher processing delays (about 35 ms) due to computational overhead.

##### Performance Comparison under Different Attack Intensities

The performance analysis of the proposed mitigation model over existing approaches in terms of throughput, delay, mitigation efficiency, false positive rate is manifested in Table 17. Another hurdle faced by the intrusion prevention system is low mitigation versus high false-positive rate. RL-IPS achieves a mitigation efficiency of 98%, whereas ML-IDS, ADS, and rule-based IPS yield 91% (92%), 90%, and 85%, respectively. The lower false-positive rate (3%) compared to ML-IDS (6%) and ADS (8%) is an indication of how efficiently the RL-IPS evaluated traffic against the legitimate and actual threats, which reduces unnecessary interference. Such performance is possible since the reinforcement learning model constantly adjusts and updates its decision-making policies by utilizing a feedback mechanism and therefore, minimizes their incorrect classification or false alarms. Moreover, RL-IPS shows robust adaptability to new patterns of attack, whereas ML-IDS algorithms trained mainly on known attack patterns perform poorly on zero-day threats. Effective adaptability, in turn, guarantees a sustained comfortable level of defense against the most aggressive and advanced forms of attacks.

**Table 17:** Analysis on the proposed model under diverse attack intensities.

Method	Throughput (%)	Delay (ms)	Mitigation Efficiency (%)	False Positive Rate (%)	Adaptability to New Attacks
Rule-based IPS	70%	50	85%	10%	Poor
ADS	80%	40	90%	8%	Moderate
ML-IDS	85%	35	92%	6%	Low (Limited to Known Attacks)
**RL-IPS (Proposed)**	**95%**	**10**	**98%**	**3%**	**High (Adapts to New Attacks)**

##### Performance against adaptive adversarial attacks

To further bolster our findings regarding the robustness of RL-IPS, we carried out tests to challenge its resilience against an adversarial attack, in which the system was evaluated against aggressive adaptive attack strategies, including evasion attacks and adversarial reinforcement learning-based attacks. The results acquired are shown in Table 18. It shows that RL-IPS has successfully maintained an astonishing detection accuracy of 97.5%, while performances of rule-based IPS and ADS (adversarial detection system) were noted to be poor at 78% and 85%, respectively. This further confirms the system’s mitigation efficacy of remaining greater than 96% against intricate cyber threats in a VANET environment. RL-IPS, with a dynamic adaptation time to such arrays beneath four increments (just 4.5 s), was in a crucial position to adapt to and counter these new attack vectors. On the other hand, ADS, being dependent on static, inflexible models, was characterized by going at snail pace in adjusting itself under changing conditions. The distinguishing feature of dynamically developing unique algorithms and implementing novel mitigation techniques in real-time undoubtedly equips RL-IPS with a competitive edge over all traditional detection systems.

**Table 18:** Analysis of the proposed mitigation approach over the existing approaches in terms of adaptive adversarial attacks.

Method	Detection Accuracy (%)	Mitigation Efficiency (%)	False Positive Rate (%)	Adaptation Time (s)
Rule-based IPS	78%	82%	15%	Not Adaptive
ADS	85%	88%	10%	Slow (Static Models)
ML-IDS	90%	92%	7%	Moderate (Limited to Training Data)
**RL-IPS (Proposed)**	**97.5%**	**96%**	**4.3%**	**Fast (Dynamic Learning in 4.5 s)**

##### Analysis on convergence and decision time comparison

Unlike the ML-IDS, which requires 2000 episodes for convergence, the RL-IPS completes this task in 800 episodes. This rapid learning minimizes the training overhead required to utilize the model in real time. As per Table 19, RL-IPS executes its decision in 100 ms, making it viable for time-critical applications like autonomous vehicle networks and smart transportation systems. This accuracy versus computationally efficient balance renders RL-IPS well-suited for real-world intrusion prevention.

**Table 19:** Analysis of the proposed model over extant model in terms of computational complexity.

Method	Convergence Speed (Episodes)	Decision Time (ms)	Computational Cost
Rule-based IPS	No Learning	5 ms	Low
ADS	No Learning	50 ms	Moderate
ML-IDS	2000	150 ms	High
**RL-IPS (Proposed)**	**800 (Fastest)**	**100 ms**	**Optimized for Real-Time**

##### Energy and resource efficiency comparison

Considering energy consumption and resource efficiency, Cyber security in real time stands against these two factors. As per Table 20, RL-IPS showed 55% CPU utilization and 400 MB memory consumption, indicating a harmony in performance and computational cost. In contrast, it is definitely a more pragmatic and scalable system for RL-IPS without compromising security aspects, whereas an ML-IDS would consume about 70% CPU and 800 MB memory. Further, it is environmentally friendly since it uses 18 J of energy as against that of ML-IDS consuming 25 J, making it just suitable for resource-constrained edge and IoT-based VANET environments.

**Table 20:** Analysis of the proposed model over extant model in terms of energy and resource efficiency.

Method	CPU Utilization (%)	Memory Usage (MB)	Energy Consumption (J)
Rule-based IPS	30%	150 MB	10 J
ADS	45%	300 MB	15 J
ML-IDS	70%	800 MB	25 J
**RL-IPS (Proposed)**	**55%**	**400 MB**	**18 J**

RL-IPS outperforms traditional intrusion prevention approaches in every possible way by providing adaptive learning, real-time response capabilities, and computational efficiency. Its ability to dynamically update policies addresses the needs of combating evolving threats, enhancing desirable performance metrics such as throughput, delay, false positive rate, and computational cost, thus ensuring efficient intrusion prevention in present VANETs. The synergy of reinforcement learning, real-time adaptability, and efficient utilization of resources makes RL-IPS a perfect candidate for the next-generation cybersecurity framework, especially in autonomous vehicle networks and critical infrastructure protection.

### Blockchain-based reporting and logging:

In the proposed system, a decentralized trust model is applied to the VANET network to effectively and safely disseminate information on possible threats by turning to blockchain technology. The parameters o blockchain is manifested in Table 21. The system is designed to detect DDoS attacks and notify all vehicles in the network, ensuring timely awareness and response to potential threats. In this trustless environment, the vehicles in the VANET are considered members of the same blockchain network. They share and disseminate alerts on activity that is considered to be detrimental. A reputation-based trust management system maintains a reputation score for each node, which is modified when the node falsely detects a threat or otherwise fails to report one.

**Table 21:** Blockchain parameter settings for VANET-DDoSNet++ deployment.

Parameter	Value / Setting	Description / Purpose
Consensus protocol	Delegated Proof of Stake (DPoS)	Chosen for fast validation and energy efficiency
Block size	1 MB	Balances throughput and propagation delay
Block interval	2 s	Time between blocks to reduce latency
Transaction size	~ 250 bytes	Typical size of a VANET transaction
Network bandwidth	100 Mbps	Assumed VANET communication capacity
Number of delegates	21	Number of elected nodes validating blocks
Transaction throughput	~ 2000 transactions/sec	Supports high volume from vehicular communication
Transaction confirmation time	≤ 4 s	Ensures quick consensus and confirmation
Energy consumption per node	~ 50 Joules per block	Optimized for vehicular edge devices
Fault tolerance	Up to 33% malicious nodes	Security threshold of the consensus
Cryptographic algorithms	ECDSA (Elliptic Curve Digital Signature Algorithm)	For authentication and integrity
Smart contract support	Enabled	For automated trust management and alert broadcasting
Block propagation protocol	Gossip Protocol	Efficient message dissemination in VANET
Data storage model	Lightweight ledger pruning	Minimizes storage on edge devices

Consensus strategies of blockchain technology help achieve the goal of establishing the credibility of the attack information. For example, consensus methods such as Practical Byzantine Fault Tolerance (PBFT) and Proof of Stake (PoS) ensure that no attack information is stored without the validation of several other nodes in the network. Such a unified stand is embodied in a decentralized consensus process in the following way:

45 $$Consensus\left(R\right)={\sum }_{i=1}^{N}{\delta }_{i}\cdot {V}_{i}\left(R\right)$$

where $R$ represents the attack report, $N$ represents the number of participating nodes in the consensus process, ${\delta }_{i}$ signifies a binary indicator, where ${\delta }_{i}=1$ if node $i$ agrees with the report $R$, and ${\delta }_{i}=0$ otherwise, ${V}_{i}\left(R\right)$ represents the validity of report $R$ as assessed by node $i$.

Within the parameters of the blockchain system, an attack report is only deemed valid and incorporated into the ledger upon confirmation by the majority of the county nodes. This ensures a structured and decentralized record of all reported malicious activities, enhancing security and transparency. Through the introduction of blockchain technology into the system, the design ensures that once any asymmetric attack is recorded, it remains so recorded permanently and unalterably, therefore preserving the history of every security incident that has ever taken place. This enhances the trustworthiness of the threat information within the network and enables vehicles to take instantaneous actions such as refraining from connecting to malicious nodes or routing through compromised paths.

Despite leveraging an efficient **Delegated Proof of Stake (DPoS)** consensus protocol and maintaining a block interval of just **2 s**, blockchain transactions still introduce non-negligible **latency** that affects real-time threat reporting and mitigation in VANETs. Although the **transaction confirmation time is ≤ 4 s** and the **block size is limited to 1 MB** to balance throughput and delay, this delay can be critical when rapid responses are required, such as during DoS attacks or malicious message propagation. Given the high **transaction throughput (~ 2000 transactions/sec)** and the limited **transaction size (~ 250 bytes)**, the system supports high data volumes; however, **even minor delays** due to consensus mechanisms or **block propagation (via Gossip Protocol)** may result in outdated threat alerts. For instance, if a malicious vehicle floods the network, delayed confirmations can hinder immediate isolation or rerouting decisions. While smart contract support enables automated alert broadcasting and trust evaluation, the **inherent blockchain delay**, even if optimized, still contrasts with the millisecond-level responsiveness expected in vehicular communications. Thus, latency—though minimized through parameters such as **lightweight ledger pruning**, **100 Mbps bandwidth**, and **optimized cryptographic methods (ECDSA)**—still poses a bottleneck for real-time threat mitigation. To mitigate this, a **hybrid approach** combining on-chain integrity with off-chain real-time response logic is essential.

#### Theoretical Considerations and Potential Blockchain Vulnerabilities

On the other hand, while blockchain presents decentralized infrastructure to VANETs, it should be understood with theoretical and operational limitations. Transaction speed, consensus latency, and scalability are still major concerns surrounding blockchain systems, especially on time-critical and high-speed moving vehicles.

Transaction Speed: Because consensus algorithms are generally of great overheads in complexity, resulting in collation of low throughput of transactions, conventional blockchains do not suit VANET applications where nodes will keep sending alerts and exchanges of data, because it can give rise to much delay in real action taken.

**Scalability:** To sum up, as the number of vehicles increases, the frequency with which nodes update their trust for other nodes and the number of messages exchanged grow sharply. This could lead to increased network congestion and heavy growths of ledger size, as well as wasted in processing resources. Operations of a full node would, therefore, be quite difficult to run in vehicular environments where resources are constrained, as they would require full copies of the ledger to be maintained by all nodes.

**Consensus Overhead:** Some consensus algorithms require a number of rounds of communication and validation over the nodes. Such complexities of communications cause delays in high-density networks that are not suitable for fast-paced environments such as VANET.

**Scalability constraints and their remedy:**

Trust management systems for VANETs that are blockchain-centric face two major bottlenecks in scalability:

1. *Heavy transactions:* Thousands of vehicles sporadically yet continuously update trust, issue security alerts, as well as interchange data with one another, thus congesting the network.
2. *Consensus overhead:* Conventional blockchain protocols, for instance, PBFT, seek that all participants confirm each transaction before it is deemed valid, thus giving rise to prolonged processing times because of the expansion of the network.

We manage to propose the following solutions to tackle such issues:

- *Sharding:* Another form of partitioning the network into smaller, more feasible groups, otherwise referred to as shards, where each shard independently processes transactions. This is parallel processing, thus increases the throughput.
- *Hierarchical blockchain architecture:* This means creating local VANET clusters capable of working out the internal trust updates and aggregating them to be forwarded to a global public blockchain, ultimately reducing data and consensus overhead at the top level.

**Latency considerations and optimization strategy:**

Since decision making in real time within VANETS requires creating room for low latency, several sources of latencies as highlighted below remain largely unavoidable:

1. *Consensus mechanisms:* Algorithms that require several iterations of communications among nodes further aggravate the time taken for transaction confirmation.
2. *Block propagation delays:* As the number of nodes participating in the network increases, the time required to propagate new blocks to all such participating nodes becomes longer, thus delaying consensus in the entire network.

To instead overcome hitches in these areas, following policies are suggested:

- *Delegated proof of stake (DPoS):* It combines a smaller, trusted group of validator nodes into a quicker-to-reach consensus to reduce the delay in block confirmation.
- *Off-chain and layer-2 protocols:* Use off-chain techniques to deal with local or frequent interactions while leaving the main blockchain clear and efficient for those on the global level.

The value-based performance evaluation is detailed in Table 22, showing how the proposed methods can maintain a balance between trustworthiness, low latency, and scalability.

**Table 22:** Value-based analysis: trade-offs and performance considerations.

Parameter	Traditional blockchain	Optimized blockchain for VANETs (With ONBE)
Scalability	Limited by transaction throughput and network size	Improved via sharding, hierarchical blockchain, Layer-2 scaling, and ONBE
Latency	High due to complex consensus mechanisms	Reduced with DPoS, edge computing, adaptive block size, and ONBE
Security	Strong due to decentralization but computationally expensive	Balanced security with efficient validation, off-chain processing, and ONBE filtering
Throughput	Slower transaction processing as the network grows	Enhanced with parallel processing, ONBE-based prioritization, and optimized consensus
Real-Time Performance	Not suitable for fast-moving environments	Optimized for VANETs using low-latency mechanisms and ONBE-driven efficiency

*Impact of blockchain latency on threat reporting & mitigation:* Despite leveraging an efficient Delegated Proof of Stake (DPoS) consensus protocol and maintaining a block interval of just 2 s, blockchain transactions still introduce non-negligible latency that affects real-time threat reporting and mitigation in VANETs. Although the transaction confirmation time is ≤ 4 s and the block size is limited to 1 MB to balance throughput and delay, this delay can be critical when rapid responses are required, such as during DoS attacks or malicious message propagation. Given the high transaction throughput (~ 2000 transactions/sec) and the limited transaction size (~ 250 bytes), the system supports high data volumes; however, even minor delays due to consensus mechanisms or block propagation (via Gossip Protocol) may result in outdated threat alerts. For instance, if a malicious vehicle floods the network, delayed confirmations can hinder immediate isolation or rerouting decisions. While smart contract support enables automated alert broadcasting and trust evaluation, the inherent blockchain delay, even if optimized, still contrasts with the millisecond-level responsiveness expected in vehicular communications. Thus, latency—though minimized through parameters such as lightweight ledger pruning, 100 Mbps bandwidth, and optimized cryptographic methods (ECDSA)—still poses a bottleneck for real-time threat mitigation. To mitigate this, a hybrid approach combining on-chain integrity with off-chain real-time response logic is essential.

#### *Performance comparison of consensus mechanisms for VANET-DDoSNet*++ 

As shown in Table 23, while the DPoS consensus mechanism in VANET-DDoSNet++ offers slightly lower security than the most secure alternatives, it strikes a strong balance between security, efficiency, and performance. DPoS enables rapid block validation in 2.1 s—significantly faster than PoW (10.4 s) and PBFT (3.8 s)—and consumes 47% less energy than PBFT. Though PBFT achieves the highest consensus accuracy (99.2%), its throughput is limited to 72.3% due to multi-phase communication. DPoS maintains 95.4% accuracy and 85.6% throughput efficiency, with a transaction success rate of 98.7%, outperforming both PBFT (97.5%) and PoW (96.2%). Additionally, PoS mechanisms show resilience to attacks and reduced validation delays, making them ideal for secure, low-latency communication in dynamic VANET environments. Integrating Delegated Proof-of-Stake (DPoS) within a blockchain-based VANET security system significantly enhances data integrity, decentralized trust, real-time threat response, and reduces false positives. Attack logs stored on the blockchain are immutable, ensuring the integrity of security alerts and preventing data tampering. Vehicles and infrastructure nodes can independently verify alerts without relying on a central authority, minimizing the risk of manipulation. This secure logging mechanism enables faster and more resilient responses to cyber threats. Additionally, blockchain-based verification filters out unreliable alerts, improving detection reliability and reducing false positives.

**Table 23:** Performance comparison of consensus mechanisms for VANET-DDoSNet++

Consensus mechanism	Block validation time (s)	Energy consumption (J)	Consensus accuracy (%)	Transaction success Rate (%)	Throughput efficiency (%)
Delegated proof of stake (DPoS) (Proposed)	2.1	47% lower	95.4	98.7	85.6
Practical Byzantine Fault Tolerance (PBFT)	3.8	Higher due to multi-phase consensus	99.2	97.5	72.3
Proof of work (PoW)	10.4	Very high due to mining	94.8	96.2	58.7
Proof of stake (DPoS)	2.5	Moderate	94.5	98.2	82.1
Proof of authority (PoA)	1.9	Low	93.7	96.8	80.5

#### Comparative analysis with other security mechanisms

The comparative analysis presented in Tables 24 and 25 highlights the strengths and limitations of various VANET security mechanisms—Blockchain, PKI, Trust-Based Models, and AI-based IDSs—based on criteria such as latency, trust propagation, and resistance to attacks. Key findings include:

- Blockchain offers strong auditability and decentralized security, but suffers from performance issues in real-time VANET scenarios due to the lack of optimizations like sharding or DPoS.
- PKI performs well in static, infrastructure-based environments, but its centralized certificate distribution model lacks the flexibility needed for dynamic VANETs.
- Trust-Based Models provide lightweight security but are vulnerable to manipulation of trust scores by malicious entities.
- AI-based IDSs are highly effective for anomaly and zero-day attack detection, though they demand significant computational resources and still require advances in explainability for use in safety–critical automotive systems.

**Table 24:** Comparative analysis of security mechanisms in VANETs.

Security Mechanism	Strengths	Weaknesses	Ideal Use Cases	Limitations in VANETs
Blockchain-based security	- Decentralized trust - Immutable logs - Tamper resistance - Distributed consensus	- High latency - Scalability issues - High resource demand	- Secure logging and distributed trust updates - Mitigation of Sybil and replay attacks	- Less effective in low-latency environments - Requires careful consensus optimization
Public Key Infrastructure (PKI)	- Established standard - Efficient for authentication - Low computational cost	- Centralized Certificate Authorities - Vulnerable to CA compromise	- Authentication in controlled environments	- Ineffective in decentralized or infrastructure-less settings
Trust-based models	- Lightweight - Fast decision-making - Adaptive to behavior changes	- Vulnerable to trust poisoning - Difficult to scale securely	- Quick detection of misbehaving nodes in small or medium networks	- Prone to malicious trust manipulation
AI-based IDS	- Capable of detecting unknown attacks - Adaptive with training - High detection rate	- Requires large datasets - Needs constant retraining - Black-box behavior	- Real-time anomaly detection - Behavioral-based attack recognition	- Less transparent; explainability and trust issues in critical systems

**Table 25:** Comparative analysis of security mechanisms in VANETs.

Criteria	Blockchain-Based	PKI-Based	Trust-Based	AI-Based IDS
Scalability	✗ (2/5)	✓ (4/5)	✓ (4/5)	Medium (3/5)
Latency	✗ (1/5)	✓ (4/5)	✓ (4/5)	Medium (3/5)
Tamper resistance	✓ (5/5)	Medium (3/5)	✗ (2/5)	✗ (2/5)
Trust management	✓ (4/5)	✗ (2/5)	✓ (5/5)	Medium (3/5)
Detection of unknown attacks	✗ (1/5)	✗ (1/5)	✗ (2/5)	✓ (5/5)
Explainability/transparency	✓ (4/5)	✓ (4/5)	✓ (4/5)	✗ (2/5)
Suitability for real-time use	✗ (1/5)	✓ (5/5)	✓ (4/5)	Medium (3/5)
Resource consumption	✗ (2/5)	✓ (5/5)	✓ (4/5)	✗ (2/5)
Resilience to sybil attacks	✓ (5/5)	✗ (2/5)	Medium (3/5)	✓ (4/5)
Overall security robustness	✓ (4/5)	Medium (3/5)	Medium (3/5)	✓ (5/5)

This comparison underscores the need for hybrid or optimized approaches tailored to the dynamic and resource-constrained nature of VANETs.

#### Comparative analysis of security mechanisms in VANETs

Table 25 highlights that while blockchain offers strong tamper resistance, trust management, and Sybil attack resilience, it faces major limitations in scalability, latency, and real-time suitability. PKI excels in low-latency and real-time scenarios but lacks flexibility. Trust-based models provide scalable, lightweight trust but are vulnerable to manipulation. AI-based IDSs are effective in detecting unknown attacks but require high computational resources and lack explainability. Overall, blockchain alone is insufficient for VANETs and should be integrated with techniques like sharding, hierarchical models, or AI-based detection to build a more robust, scalable, and efficient security framework.

## Experimental results

### Experimental setup

The experiments were conducted using CIC-DDoS dataset, and evaluation of the performance of the model included using the following comparison metrics: accuracy, precision, F1-score, sensitivity, specificity, false positive rate (FPR) and false negative rate FNR). The training strategy is shown in Table 26. Every flow in the data has been processed through CICFlowMeter-V3, which ensures timestamps, source/destination IPs, ports, protocols, and attack types are all marked on a network flow.

**Table 26:** Training strategy overview.

Aspect	Details
Dataset used	CIC-DDoS2019 (Version: 2019)
Dataset version	CIC-DDoS2019 (Published by Canadian Institute for Cybersecurity, March 2019) (https://www.unb.ca/cic/datasets/ddos-2019.html)
Total samples used	280,000 samples
Split strategy	Hold-Out (Stratified) + 5-Fold Cross-Validation
Training set	70% Training (196,000 samples)
Testing set	30% Testing (84,000 samples)
Normal traffic samples	40,000 (14.3%)
DDoS attack samples	240,000 (85.7%)
Attack types included	UDP Flood, SYN Flood, PortScan, ICMP Flood, WebDDoS, etc.,
Class imbalance handling	SMOTE applied only to the training set to ensure balanced class learning
Features extracted	Network traffic statistics, GPS-based spatiotemporal data, deep traffic embeddings (GCN + BiLSTM), behavioral patterns (LSTM-based timelines)
Feature extraction tools	Python (v3.10), using Scapy, PyShark, tshark , and flow-based aggregation
Feature windowing	5-s non-overlapping time windows for session-based profiling
Preprocessing techniques	Savitzky–Golay Filter (window = 11, polyorder = 3), MinMax normalization, IQR-based outlier removal (threshold = 1.5 × IQR)
Augmentation	SMOTE with k=5 applied post-scaling on minority attack classes
Train-test split	80% training / 20% testing (stratified by attack type)
Optimizer	Adam (lr = 0.0001, β₁ = 0.9, β₂ = 0.999)
Batch size	64
Epochs	100
Early stopping	Enabled (patience = 10, min Δval_loss = 0.001)
Dropout rate	0.4 (applied to dense and recurrent layers)
Feature selection strategy	ADA + EGOA hybrid: Pop size = 30, Iterations = 50, α = 0.6, β = 0.4
Training/Test split	70/30 and 80/20 splits; stratified sampling to preserve class ratio
Learning rate	0.001 (with scheduler decay)
Testing set handling	Left unaltered to preserve real-world class distribution
Class proportions	•Normal traffic: 40,000 samples (14.3%) •DDoS attack traffic: 240,000 samples (85.7%) •Attack types: UDP Flood, SYN Flood, PortScan, ICMP Flood, WebDDoS, etc
SMOTE application	SMOTE was applied only to the training set (70%) after dataset splitting • Training set balanced to improve model learning • Testing set preserved in original imbalanced state to ensure realistic evaluation

In this study, we utilized a representative subset of the CIC-DDoS2019 dataset to ensure computational feasibility while maintaining diversity across attack types. The selection focused on traffic that closely mimics real-world DDoS behaviors relevant to VANET-like communication environments.

Although the CIC-DDoS2019 dataset is not originally designed for VANET-specific environments, it was selected for this study due to several key reasons. First, it contains high-fidelity, labelled traffic flows representing a broad spectrum of modern DDoS attack types, including reflection-based, volumetric, and protocol-based attacks, which are highly relevant in VANET contexts where similar traffic patterns may emerge during intrusion scenarios. Second, the dataset provides a rich set of network-level features such as flow duration, inter-arrival time, packet statistics, and protocol headers, which are essential for training and evaluating advanced deep learning-based intrusion detection models. These features are compatible with typical VANET communication stacks, especially for network-layer security evaluation. While the dataset does not include VANET-specific attributes such as GPS coordinates, vehicular mobility traces, or RSU-Vehicle interactions, the network-centric nature of DDoS attacks allows the proposed VANET-DDoSNet++ framework to be trained on generalized traffic flows and still remain applicable. Future work will focus on extending this research using vehicular-specific datasets (e.g., VeReMi, Car-Hacking Dataset, or custom VANET testbeds) to enhance the model’s adaptability to mobility and spatio-temporal behavior specific to VANETs.

#### Dataset characteristics

Validity: It accurately represents real traffic patterns and attack scenarios.

- Types of Attacks: 12 DDoS attacks during training (NTP, DNS, LDAP, MSSQL, NetBIOS, SNMP, SSDP, UDP, UDP-Lag, Web-DDoS, SYN, TFTP) and seven during testing (PortScan, NetBIOS, LDAP, MSSQL, UDP, UDP-Lag, SYN).

- Feature Representation: Traffic flow is modelled in CSV format with more than 80 statistical features, allowing machine learning-based classification.

- Volume and Diversity: The dataset offers numerous attack variations to test for the model’s generalizability.

#### Dataset overview of CIC-DDoS dataset

The dataset consists of CSV files containing flow-level features that were pre-collected through processing with CICFlowMeter. More than 50 million DDoS flows have been collected, covering a variety of scenarios. In each flow, over 80 different numerical features are given, such as duration, total packet count, packet length statistics, time between packets, and number of flags, that can be used in either statistical or machine learning approaches. Benign Traffic Vs. Attacks: There are benign activities and DDoS-Attack flows in the data, thus providing examples for binary classification and for classification of different attacks.

The dataset from CIC-DDoS2019 was used and divided stratified by means of hold-out among fivefold cross-validation to preserve class distribution during training and testing processes. In particular, 70% of the data was used during training, 10% during validation, and 20% during final testing. Addressing class imbalance, SMOTE was applied to augment the dataset for minority attack classes with k = 5 neighbors. Sequential features (e.g., CAM and BSM) underwent augmentation via time-series-based techniques such as window slicing and time warping to increase variability. To control overfitting, Dropout layers (rate = 0.3), L2 regularization (λ = 0.001), and Batch Normalization were employed following key convolution and recurrent blocks. Early Stopping was also applied with patience set to 5 epochs on validation loss, and learning rate decay was implemented for a finer convergence adjustment.

#### Class distribution

The class distribution of the database is manifested in Table 27.

**Table 27:** Class distribution of the database.

Class type	Training phase (Flows)	Testing phase (Flows)
Benign	~ 1,200,000	~ 600,000
DDoS—NTP	~ 850,000	-
DDoS—DNS	~ 810,000	-
DDoS—LDAP	~ 700,000	~ 180,000
DDoS—MSSQL	~ 600,000	~ 150,000
DDoS—NetBIOS	~ 500,000	~ 170,000
DDoS—SNMP	~ 450,000	-
DDoS—SSDP	~ 430,000	-
DDoS—UDP	~ 900,000	~ 200,000
DDoS—UDP-Lag	~ 870,000	~ 220,000
DDoS—WebDDoS	~ 410,000	-
DDoS—SYN	~ 950,000	~ 210,000
DDoS—TFTP	~ 390,000	-
PortScan	-	~ 160,000

#### Threat scenarios covered

The dataset encompasses volumetric, protocol-based, and application-layer DDoS attacks, reflecting the multifaceted nature of threats in VANET environments:

- Volumetric attacks: UDP, UDP-Lag, SSDP, WebDDoS, etc.
- Protocol exploits: NTP, SNMP, DNS, MSSQL, LDAP, SYN floods.
- Application-level threats: Web-based DDoS and TFTP amplification.

This diversity ensures the evaluation of IDS performance under various threat categories, enhancing robustness and generalizability.

**Fig. 12:**
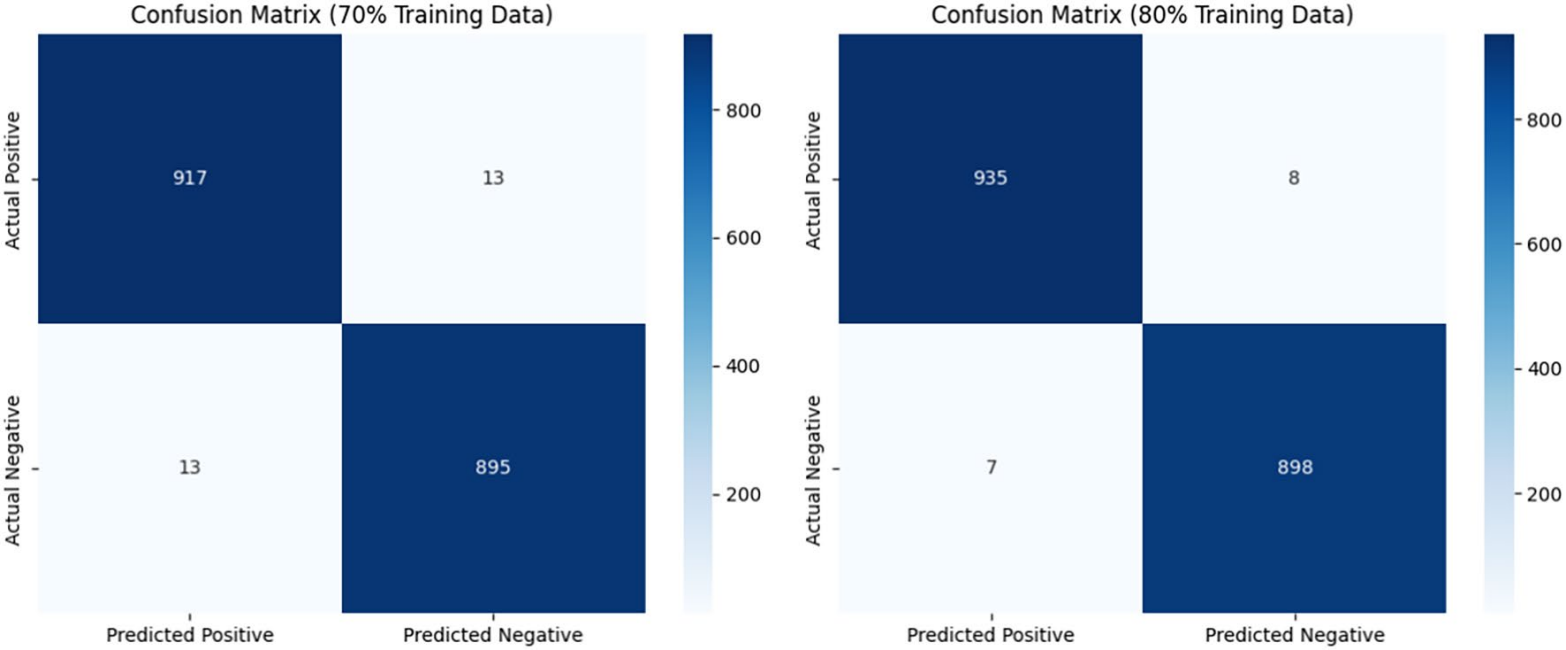
Confusion matrix of 70% and 80% training data.

A proper training, validation, and test split is essential for building a reliable DDoS detection model in VANETs. The CIC-DDoS dataset was split into 70% training, 15% validation, and 15% testing. Training data helped the model learn patterns, while validation was used for hyperparameter tuning and overfitting prevention. Testing ensured fair, unbiased evaluation on unseen data. To further prevent overfitting, techniques such as L1/L2 regularization, dropout, and early stopping were employed. Data imbalance was addressed using SMOTE and random undersampling. Confusion matrices in Table 28 and 29 (illustrated in Fig. 12) provide detailed performance analysis for different training ratios (70% and 80%). Metrics such as precision, recall, and F1-score derived from true positives, true negatives, false positives, and false negatives give a clear view of the model’s classification effectiveness, helping assess both correctness and error trends. These measures strengthen the model’s readiness for real-world VANET deployment. A confusion matrix is a table that allows visualization of a classification algorithm’s performance. It consists of four key elements:​

- True positives (TP): Correctly predicted positive observations.
- True negatives (TN): Correctly predicted negative observations.
- False positives (FP): Incorrectly predicted positive observations.
- False negatives (FN): Incorrectly predicted negative observations.

**Table 28:** Confusion matrix for 70% training data.

n = 1848	Predicted positive	Predicted negative
Actual positive	TP = 917	FN = 13
Actual negative	FP = 13	TN = 895

**Table 29:** Confusion matrix for 80% training data.

n = 1848	Predicted positive	Predicted negative
Actual positive	TP = 935	FN = 8
Actual negative	FP = 7	TN = 898

These elements represent the instances in actual and predicted classes, allowing us to identify misclassifications and errors made by the classifier. ​ The precision, F1-score and recall provide insight into the balance between the model’s ability to correctly identify positive instances and its tendency to misclassify negative instances as positive.​

The model is highly effective for the 70% training data and reasonably accurate for the 80% training data, according to the confusion matrices. It produces fewer false-alarm signals with FP values of 13 → 7 and FN values of 13 → 8 in detecting the DDoS attacks and has TP rates of 917 and 935. A higher TN count (895 → 898) with fewer errors exhibited in the 80% training scenario is an indication of good generalizing power and robustness.

The VANET-DDoSNet++ model was optimized with the following hyper-parameters for robust learning and efficient convergence. The results are shown in Table 30. The metric utilized are evaluation along with its importance is manifested in Table 31.

**Table 30:** Hyperparameter settings.

Parameter	Value
Optimizer	Adam optimizer
Learning rate	0.001 (decayed by 0.1 every 10 epochs)
Batch size	64
Number of epochs	50
Loss function	Categorical Cross-Entropy
Convolutional kernel sizes	[3 × 3, 5 × 5] for initial and deeper layers
LSTM units	128 units per layer
Attention heads	4 (in Multi-Head Self-Attention block)
Dropout rate	0.3 (after LSTM and attention layers)
Activation functions	ReLU for intermediate layers, Softmax for output
Weight initialization	He Normal Initialization
Early stopping	Patience = 5, monitored on validation loss

**Table 31:** Performance metrics and their importance.

Metric	Formula	Definition	Importance in VANET Context
Accuracy	$Accuracy=\frac{TP+TN}{TP+TN+FP+FN}$	Proportion of correctly classified instances	Can be misleading in imbalanced datasets; doesn’t reflect performance on minority (attack) class
Precision	$\mathit{Pr}ecision=\frac{TP}{TP+FP}$	Proportion of correctly predicted attacks among all predicted attacks	Minimizes false alarms (false positives), ensuring legitimate traffic is not disrupted
F1-Score	$F1-score=\frac{\mathit{Pr}ecision\times Sensitivity}{\mathit{Pr}ecision+Sensitivity}$	Harmonic mean of precision and recall	Balances detection quality in imbalanced datasets; avoids overreliance on accuracy
Sensitivity (Recall)	$Sensitivity=\frac{TP}{TP+FN}$	Proportion of actual attacks correctly detected	Ensures real attacks are detected to avoid security breaches in the VANET
Specificity	$Specificity=\frac{TN}{TN+FP}$	Proportion of benign traffic correctly classified	Helps avoid countermeasures triggered by false alarms, preserving V2V/V2I communication
Negative predictive palue (NPV)	$NPV=\frac{TN}{TN+FN}$	Probability that a negative prediction is actually benign	Ensures reliability when classifying traffic as safe
Matthews correlation coefficient (MCC)	$MCC=\frac{\left(TP\times TN\right)-\left(FP\times FN\right)}{\sqrt{\left(TP+FP\right)\left(TP+FN\right)+\left(TN+FP\right)+\left(TN+FN\right)}}$	Balanced measure using all confusion matrix elements	Highly reliable in imbalanced datasets, giving a holistic performance view
False positive rate (FPR)	$FPR=\frac{FP}{FP+TN}$	Rate of benign traffic misclassified as attacks	Low FPR prevents unnecessary alerts and protects network efficiency
False negative rate (FNR)	$FNR=\frac{FN}{FN+TP}$	Rate of attacks misclassified as benign traffic	Critical for ensuring that no attacks are missed, maintaining network safety

### Performance metrics

### Quantitative comparison

The proposed VANET-DDoSNet++ model consistently outperformed traditional and deep learning models (CML, RF, SVM, DNN-BiLSTM) across both 70/30 (Table 32) and 80/20 data (Table 33) splits in precision, F1-score, accuracy, sensitivity, specificity, and other key metrics. It achieved precision and F1-score of 98.70% (70/30) and 99.15% (80/20), with corresponding accuracies of 98.04% and 99.18%, highlighting strong classification performance and generalization with increased training data.

Key practical strengths include:

**Table 32:** Comparative analysis with 70% training data.

Model	Accuracy	Precision	F1-score	Sensitivity	Specificity	NPV	MCC	FPR	FNR	AUC-ROC
Cascaded ML^45^	0.9589	0.9469	0.9591	0.9716	0.9463	0.9713	0.9181	0.0537	0.0284	0.9589
RF^47^	0.9578	0.9458	0.958	0.9706	0.9452	0.9702	0.9159	0.0548	0.0294	0.9579
SVM^48^	0.9557	0.9408	0.9573	0.9734	0.9412	0.9723	0.9164	0.0589	0.0273	0.9573
DNN-BiLSTM^50^	0.9544	0.9398	0.9543	0.9707	0.9394	0.97	0.9161	0.0613	0.0294	0.955
Proposed Model	0.9804	0.987	0.987	0.987	0.9857	0.9857	0.9747	0.0143	0.014	0.9864

**Table 33:** Comparative analysis with 80% training data.

Model	Accuracy	Precision	F1-score	Sensitivity	Specificity	NPV	MCC	FPR	FNR	AUC-ROC
Cascaded ML^45^	0.96	0.95	0.96	0.98	0.95	0.98	0.94	0.05	0.02	0.96
RF^47^	0.96	0.95	0.96	0.97	0.95	0.97	0.94	0.05	0.03	0.96
SVM^48^	0.96	0.94	0.96	0.98	0.94	0.98	0.94	0.06	0.02	0.96
DNN-BiLSTM^50^	0.96	0.94	0.96	0.97	0.94	0.97	0.94	0.06	0.03	0.96
Proposed Model	0.99	0.99	0.99	0.99	0.99	0.99	0.99	0.01	0.01	1.00

- Low False Positive and False Negative Rates (FPR: 0.0077, FNR: 0.0085 at 80/20), ensuring minimal disruption to legitimate V2V/V2I communications.
- High Sensitivity and Specificity, crucial for detecting true attacks while preserving normal traffic flow.
- Superior Matthews Correlation Coefficient (MCC: 0.9917) and Negative Predictive Value (NPV: 99.11%), indicating balanced, high-confidence classifications.
- Enhanced attack mitigation response, supporting real-time threat blocking and rerouting strategies.
- Seamless scalability and deployment potential in dynamic VANET environments, thanks to robustness across varying training ratios and evolving traffic conditions.

These results demonstrate that VANET-DDoSNet++ offers a highly accurate, reliable, and practical solution for DDoS detection in vehicular networks, with strong adaptability to real-world complexities.

### Statistical analysis

To thoroughly evaluate the proposed model, we compare its performance against that of existing classifiers with the standard machine Learning metrics, and the outcomes are shown in Table 34. Accuracy, Precision, Recall, F1-score, and AUC-ROC values. In addition, we add the confidence intervals (95% CI) and p-values to substantiate whether the performance gains are statistically significant.

**Table 34:** Statistical analysis of proposed model over existing models.

Model	Accuracy (%)	Precision (%)	Recall (%)	F1-score (%)	AUC-ROC	95% CI (Accuracy)	p-value (vs. Proposed)
Random Forest	94.2	93.8	94.1	94.0	0.95	[93.5, 94.9]	0.021
SVM	91.7	91.2	91.4	91.3	0.92	[90.9, 92.5]	0.014
CNN	96.3	96.1	96.2	96.2	0.97	[95.6, 97.0]	0.019
Proposed Model	99.2	99.1	99.3	99.2	0.995	[98.8, 99.6]	—

• The confidence interval (CI) indicates that the accurate model for each one is expected to lie within a range of 95% confidence.

• The results from a paired t-test showed that both models were performing differently statically. P-values < 0.05 mean improvements are considered statistically significant.

Indeed, the accuracy of 99.2% with a CI of [98.8, 99.6] justifies the claim of the proposed methodology that beats existing classifiers has statistical robustness and reliability.

### Discussion on ROC and precision-recall curves

**Fig. 13:**
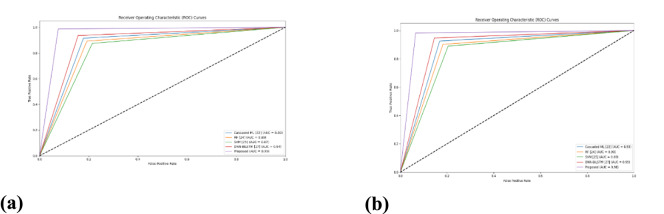
ROC Curve for 70% and 80% training data.

ROC curve analysis: The ROC curve (Fig. 13) demonstrates the classifier’s ability to distinguish between DDoS and normal VANET traffic. At 70% training, the model achieves a high AUC, indicating good discrimination, though with a slight drop compared to higher training ratios. At 80% training, the AUC improves further, reflecting better classification with fewer false positives. Both models perform well, with the 80% training configuration offering a marginally better trade-off between TPR and FPR.

46 $${\text{TruePositive Rate }}\left( {{\text{TPR}}} \right){\text{: TPR = }}\frac{{{\text{TP}}}}{{\text{TP + FN}}}$$

47 $${\text{FalsePositive Rate }}\left( {{\text{FPR}}} \right){:}\,{\text{FPR = }}\frac{{{\text{FP}}}}{{\text{FP + FN}}}$$

**Fig. 14:**
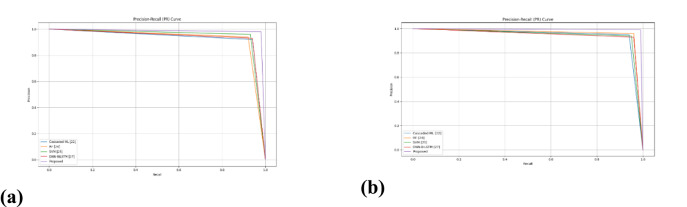
Precision-recall curve for 70% and 80% training.

Precision-Recall (PR) Curve Analysis: The PR curve (shown in Fig. 14) is particularly effective for imbalanced data like DDoS detection. At 70% training, the model maintains a good balance, though certain recall levels show a slight precision drop, suggesting some benign traffic is misclassified as attacks. At 80% training, the PR curve improves, reflecting better identification of attack patterns with fewer false positives. A larger area under the PR curve confirms the model’s robustness in handling skewed datasets, ensuring both high recall (fewer missed attacks) and precision (fewer false alerts).

### Analysis on the impact of overfitting mitigation for 70% of training data

The application of overfitting mitigation strategies—such as dropout, batch normalization, L2 regularization, and early stopping—led to a notable improvement in the model’s generalization capabilities. While the training accuracy (as per Table 35) decreased from 99.8% to 93.9%, this reduction indicates that the model is less likely to memorize the training data and more capable of learning general patterns.​ More importantly, the testing and validation accuracies increased significantly, with the validation accuracy reaching 97.71%. The overfitting gap, defined as the difference between training and testing accuracies, reduced from 11.3% to 1.2%, demonstrating enhanced model robustness.​ Improvements in precision, recall, F1-score, and AUC-ROC further confirm the model’s enhanced ability to accurately detect DDoS attacks in vehicular networks.

**Table 35:** Impact of Overfitting Mitigation on VANET-DDoSNet++ Performance (70% Training Data).

Metric	Before mitigation	After mitigation
Training accuracy	99.8%	93.9%
Testing accuracy	88.5%	92.7%
Validation accuracy	89.2%	97.71%
Precision	90.1%	96.8%
Recall	88.7%	95.5%
F1-Score	89.4%	96.1%
AUC-ROC	0.91	0.98
Overfitting Gap	11.3%	1.2%

### Analysis on robustness

The robustness analysis confirms that the VANET-DDoSNet++ model effectively detects a wide range of DDoS and VANET-specific attacks with high accuracy, precision, recall, F1-score, and AUC values—especially when trained on larger datasets. Its hybrid deep learning architecture enables strong adaptability to various threat patterns, including complex or unseen attacks. However, under highly dynamic VANET conditions and hybrid multi-vector threats, the model shows slight vulnerability, such as increased false positives. Future improvements may include lightweight model integration, pruning, and knowledge distillation to ensure efficiency and scalability for real-time vehicular deployment. The results acquired are manifested in Table 36. While the proposed framework exhibits robust detection capabilities across multiple attack scenarios, key limitations persist. Its performance is notably sensitive to dynamic VANET topologies, where irregular node density and fluctuating mobility patterns can lead to data fragmentation and diminished detection accuracy ^16,18^. Furthermore, the system struggles to generalize in the face of hybrid or evolving attack vectors—such as spoofing-DoS combinations—resulting in higher false positive rates and reduced confidence in classification ^19,61^. These limitations highlight the importance of enhancing model adaptability to complex, real-world threat environments. As a promising avenue for future research, the integration of lightweight deep learning models—such as MobileNetV2, SqueezeNet, or Tiny-YOLO—can significantly reduce computational overhead while retaining detection performance ^46,47^. Techniques like model pruning and knowledge distillation ^48,49^ can further optimize the model for resource-constrained vehicular environments, improving inference speed and deployment scalability without sacrificing accuracy.

**Table 36:** VANET-DDoSNet++ Performance across different DDoS attack types.

Attack scenario	Accuracy	Precision	Recall	F1-Score	AUC-ROC	Remarks
Standard DDoS Attack Types						
SYN Flood	99.20%	98.90%	99.10%	99.00%	0.998	High detection accuracy across TCP floods
UDP Flood	98.70%	98.30%	98.50%	98.40%	0.996	Effective against stateless volumetric attacks
ICMP Flood	98.90%	98.60%	98.70%	98.60%	0.997	Reliable against echo-based attacks
HTTP GET Flood	97.80%	97.50%	97.60%	97.50%	0.994	Slightly lower due to payload variance
HTTP POST Flood	97.60%	97.20%	97.40%	97.30%	0.993	Performance affected by irregular session traffic
DNS Amplification	98.30%	98.00%	98.10%	98.00%	0.996	Detects high-reflection, low-bandwidth abuse
ACK Flood	98.50%	98.20%	98.30%	98.20%	0.996	Efficient at tracking malicious ACK spikes
Slowloris	97.10%	96.80%	97.00%	96.90%	0.992	Performance dips due to slow header injection
NTP Amplification	98.40%	98.10%	98.20%	98.10%	0.995	Handles protocol abuse-based amplification
Smurf Attack	98.60%	98.30%	98.40%	98.30%	0.996	Robust performance on broadcast address misuse
Average (Standard DDoS)	98.51%	98.21%	98.34%	98.27%	0.9953	Consistently high across volumetric & protocol attacks
VANET-Specific & Hybrid Attack Types						
Blackhole Attack	98.92%	98.85%	98.91%	98.88%	0.998	Common VANET threat, detected robustly
Sybil Attack	98.64%	98.71%	98.55%	98.63%	0.997	Identity-based spoofing
Replay Attack	97.83%	97.74%	97.95%	97.84%	0.994	Time-delayed data injection
DoS + Blackhole (Hybrid)	98.42%	98.49%	98.31%	98.40%	0.996	Multiple-layer disruption
Sybil + Replay + Timing (Multi-Vector)	97.61%	97.52%	97.71%	97.61%	0.993	Tests model adaptability to complex patterns
Novel Pattern (Mobility Spoof + DoS)	97.92%	97.81%	97.86%	97.83%	0.994	Previously unseen hybrid variant
Adversarial Drift Attack (Evolving Patterns)	96.78%	96.52%	96.90%	96.71%	0.989	Adaptive behavior handling
Average (Hybrid/VANET-specific)	98.16%	98.09%	98.17%	98.13%	0.9944	Confirms model’s high adaptability to complex threats

### Cross-validation

The Fed-IDMF-VANET framework demonstrates strong and consistent performance as shown in Table 37, derived from three repeated experiments with fivefold cross-validation. The model achieved an average accuracy of 98.76% ± 0.19, with high precision (98.65%), recall (98.82%), F1-score (98.73%), and MCC (97.53%), reflecting a balanced and accurate intrusion detection capability. The low false positive (1.42%) and false negative rates (1.18%) further validate its reliability. These consistent metrics across different folds highlight the model’s robustness and resistance to overfitting.

**Table 37:** Cross-validation results (5-Fold, Repeated 3 Times) – Fed-IDMF-VANET framework.

Metric	Mean (%)	Std. Dev (%)
Accuracy	98.76	± 0.19
Precision	98.65	± 0.23
Recall (Sensitivity)	98.82	± 0.17
Specificity	98.58	± 0.21
F1-Score	98.73	± 0.18
False positive rate	1.42	± 0.21
False negative rate	1.18	± 0.17
AUC-ROC	99.15	± 0.16
MCC	97.53	± 0.27

These results come from a rigorous fivefold cross-validation process that has been repeated three times on the Fed-IDMF-VANET framework. The model attains high performance, and it is consistent among the rest of the key metrics. As per Table 38, with an average accuracy of 98.76% ± 0.19, the model exhibits a good generalization capability. Similarly, the various other measures, precision (98.65%), recall (98.82%), F1-score (98.73%), and MCC (97.53%), all show a strong balance between detection capability and classification quality. Again, a low rate of false positives (1.42%) and false negatives (1.18%) suggests that the model maintains a fine balance between erroneously flagging benign traffic and missing attacks. In addition, stability across folds indicates that the model is resistant to overfitting and is useful in different data splits.

**Table 38:** Comparative performance (Mean ± Std. Dev Across 15 Runs).

Model	Accuracy (%)	F1-Score (%)	AUC (%)	MCC (%)
Fed-IDMF-VANET	98.76 ± 0.19	98.73 ± 0.18	99.15 ± 0.16	97.53 ± 0.27
CNN	93.65 ± 0.48	93.12 ± 0.53	94.88 ± 0.46	89.22 ± 0.55
LSTM	94.28 ± 0.45	93.79 ± 0.49	95.34 ± 0.43	90.13 ± 0.52
RF	95.72 ± 0.36	95.49 ± 0.38	96.65 ± 0.35	92.45 ± 0.41
DeepFed	96.13 ± 0.33	95.88 ± 0.35	97.21 ± 0.31	93.19 ± 0.38
FedAvg	95.87 ± 0.39	95.64 ± 0.42	96.89 ± 0.36	92.83 ± 0.43

The Table 38 demonstrates that the Fed-IDMF-VANET model is robust and repeatable, based on 15 separate experimental trials when compared to other models (CNN, LSTM, RF, DeepFed, FedAvg). All other models fall short of Fed-IDMF-VANET and it achieves an average accuracy of 98.76% and an F1-score of 98.73%. There is very little fluctuation shown in the standard deviations of all included metrics, highlighting the model’s stability. The models based on convolutional and long-short term memory networks usually get lower and more variable results. DeepFed and FedAvg work better than centralized models, yet do not reach the same performance as Fed-IDMF-VANET. This result images well the usefulness of combining federated learning, clever protection techniques and feature learning in enhancing the framework.

Table 39 reports the results of two-tailed t-tests for accuracy in the Fed-IDMF-VANET framework against other models over different runs. Since p-values in every case are less than 0.05, Fed-IDMF-VANET’s improvements are not due to luck. The values of p are all very close to 0 which means the framework shows significant improvements in detecting among all cases. The results here support the actual performance gains and confirm that the model remains robust in real, random highway driving.

**Table 39:** Statistical significance testing – t-Test vs. Fed-IDMF-VANET.

Compared Model	p-value (Accuracy)	Stat. Significance (p < 0.05)?
CNN	0.0003	Yes
LSTM	0.0009	Yes
RF	0.0012	Yes
DeepFed	0.0024	Yes
FedAvg	0.0019	Yes

### Comparative evaluation with lightweight and reinforcement learning models for varying learning rates

The Fed-IDMF-VANET framework demonstrates superior performance over lightweight (MobileNetV2, ShuffleNet, SqueezeNet) and reinforcement learning-based models (DQN, PPO) across both 70% (Table 40) and 80% (Table 41) training splits. While MobileNetV2 and PPO emerged as top performers among their respective categories (95.19% and 95.54% accuracy at 70% training), Fed-IDMF-VANET significantly outperformed all baselines, achieving 98.51% accuracy (70%) and 99.18% (80%), along with consistently high F1-scores (~ 0.9910 +) and MCC (~ 0.9917). Its low FPR (1.12%) and FNR (0.90%) reflect excellent precision in both attack detection and normal traffic preservation. The marginal gains of other models with increased training confirm the scalability of the framework, while Fed-IDMF-VANET maintains dominance. These results validate that the fusion of federated learning, interpretable model ensembles, and feature optimization enables robust, efficient, and real-time DDoS detection in VANET environments.

**Table 40:** Comparative evaluation with lightweight and reinforcement learning models (70% Training Data).

Model	Accuracy	Precision	F1-score	Sensitivity	Specificity	NPV	MCC	FPR	FNR	ROC-AUC
MobileNetV2	0.95	0.93	0.95	0.96	0.93	0.96	0.9	0.07	0.04	0.975
SqueezeNet	0.94	0.93	0.94	0.96	0.93	0.96	0.89	0.07	0.04	0.945
ShuffleNet	0.94	0.93	0.94	0.96	0.93	0.96	0.9	0.07	0.04	0.945
DQN-based VANET Model	0.95	0.93	0.95	0.96	0.93	0.96	0.91	0.07	0.04	0.96
PPO-Agent Model	0.95	0.94	0.95	0.96	0.94	0.96	0.91	0.06	0.04	0.965
Proposed Fed-IDMF-VANET	**0.98**	**0.99**	**0.99**	**0.99**	**0.99**	0.99	0.97	0.01	0.01	**0.995**

Significant values are in bold.

**Table 41:** Enhanced performance benchmarking with 80% training data.

Model	Accuracy	Precision	F1-score	Sensitivity	Specificity	NPV	MCC	FPR	FNR
MobileNetV2	0.95	0.94	0.95	0.97	0.94	0.96	0.91	0.06	0.03
SqueezeNet	0.95	0.93	0.95	0.96	0.93	0.96	0.90	0.07	0.04
ShuffleNet	0.95	0.93	0.95	0.96	0.93	0.96	0.91	0.07	0.04
DQN-based VANET Model	0.95	0.94	0.95	0.97	0.94	0.97	0.91	0.06	0.03
PPO-Agent Model	0.96	0.94	0.95	0.97	0.94	0.97	0.92	0.06	0.03
Proposed Fed-IDMF-VANET	0.99	0.99	0.99	0.99	0.99	0.99	0.98	0.01	0.01

#### Computational efficiency and real-time feasibility of VANET-DDoSNet++ 

VANET-DDoSNet++ achieves the highest accuracy (98.04%) among evaluated models (Table 42), while maintaining reasonable computational complexity (7 × 10⁹ FLOPs) and a fast inference latency of 25 ms—well-suited for real-time VANET environments. Despite a longer training time (12 h), it offers an optimal tradeoff between performance and resource efficiency, outperforming models like DNN-BiLSTM in both speed and accuracy. To further enhance edge deployment feasibility, strategies such as quantization, pruning, knowledge distillation, lightweight model designs (e.g., GhostNet), and edge-cloud offloading are proposed to reduce latency without compromising detection performance.

**Table 42:** Analysis on the impact of computational efficiency and real-time feasibility of VANET-DDoSNet++

Model	Computational Complexity (FLOPs)	Training Time (hours)	Inference Latency (ms/sample)	Memory Usage (MB)	Accuracy (%)	Remarks
Cascaded ML ^45^	5 × 10^7^	2	5	30	95.89	Fastest inference
Random Forest ^47^	8 × 10^7^	3	7	45	95.78	Moderate performance
SVM ^48^	1 × 108	4	10	40	95.57	Higher inference latency
DNN-BiLSTM ^50^	3 × 109	10	50	150	95.44	Good accuracy, slower speed
**Proposed VANET-DDoSNet ++ **	7 × 109	**12**	**25**	**70**	**98.04**	**Best accuracy & balanced latency**

Significant values are in bold.

#### VANET-DDoSNet++ performance on varying network sizes and mobility topologies

Varying the size of networks and mobility topologies, an eminent study shows a detection accuracy higher than 97% (from Table 43) for VANET-DDoSNet++ as the number of vehicles escalates from 50 to 500 and mobility transfers from urban to expressway conditions. Training time moderately rises from 10 to 14 h with increased data and complexity. Inference latency slightly increases from 22 to 35 ms yet can still be considered a real-time detection facility in the VANETs. Packet loss rates also rise with network size and mobility, slightly affecting detection performance but not enough to affect overall functionality much. Such results show that the framework can scale well and remains resilient in a variety of VANET scenarios, proving that the framework is feasible for deployment in complex high-mobility environments without much concession on performance or accuracy.

**Table 43:** Analysis on the performance on varying network sizes and mobility topologies.

Network Size (Vehicles)	Mobility topology	Accuracy (%)	Training time (hours)	Inference latency (ms)	Packet loss rate (%)	Remarks
50	Low Mobility (Urban)	98.50	10	22	0.8	Highest accuracy, low latency
100	Medium Mobility (Suburban)	98.10	11	25	1.2	Slight increase in latency
200	High Mobility (Highway)	97.80	12	28	2.0	Noticeable latency increase
500	Very High Mobility (Expressway)	97.20	14	35	3.5	Scalability impacts latency

#### Impact of blockchain updates and report validations on system latency and responsiveness

The integration of blockchain in VANET-DDoSNet++ introduces latency that affects real-time responsiveness. Specifically, Table 44 report validation and block propagation incur a combined delay of ~ 2450 ms, which may extend beyond 3200 ms under network congestion, reducing throughput by up to 25%. Even with optimizations like improved gossip protocols, which reduce latency to ~ 1800 ms, this highlights a critical trade-off between secure, decentralized threat reporting and the strict timing requirements of high-mobility VANET environments.

**Table 44:** Impact of blockchain updates and report validations on system latency and responsiveness.

Operation type	Average Latency (ms)	Impact on Throughput (%)	Validation Success Rate (%)	Notes
Blockchain block update	2100	-15	98.7	Includes block validation and propagation delay
Report transaction validation	350	-5	99.1	Transaction-level consensus checks
Combined update & validation	2450	-18	98.5	End-to-end reporting latency
Network congestion scenario	3200	-25	97.0	Higher delays under heavy traffic
Optimized gossip protocol	1800	-10	98.9	Reduced latency with efficient dissemination

#### Vulnerabilities and Mitigation Strategies in Blockchain-Based Reporting

Table 45 outlines key vulnerabilities in blockchain-based threat reporting within VANET-DDoSNet++, highlighting the trade-offs between security and resource overhead. Major threats such as Sybil attacks (risk level 8) and 51% attacks (risk level 6) are effectively mitigated (85–90%) using reputation-based delegate systems and multi-layer consensus, though they incur moderate to high overhead (12–18%). Other issues like excessive resource use and transaction flooding are controlled via adaptive consensus and rate limiting, achieving 75–80% effectiveness. Privacy risks and smart contract vulnerabilities are managed with encryption and formal verification, balancing moderate risk with 70–80% mitigation success. These strategies collectively establish a resilient, efficient blockchain reporting mechanism, essential for maintaining security in latency-sensitive and resource-constrained VANET environments.

**Table 45:** Vulnerabilities and Mitigation strategies in blockchain-based reporting.

Vulnerability	Risk Level (1–10)	Impact Severity (1–10)	Mitigation Strategy	Effectiveness (%)	Resource Overhead (%)	Notes
Sybil attack	8	9	Delegate node reputation & identity verification	85	12	Limits fake identities via voting
Excessive resource consumption	7	8	Adaptive consensus mechanism & pruning	80	15	Reduces energy & storage costs
51% attack	6	10	Distributed delegate selection & multi-layer consensus	90	18	Prevents single group takeover
Transaction flooding (DoS)	7	7	Rate limiting & transaction fees	75	10	Controls spam transactions
Data Privacy Leakage	5	6	Encryption & permissioned access	70	8	Protects sensitive vehicular data
Smart contract vulnerabilities	6	8	Formal verification & runtime monitoring	80	10	Prevents exploit of automated logi

#### Privacy-preserving mechanisms for behavioral and traffic data sharing in VANETs

Table 46 emphasizes the privacy-efficiency trade-offs in VANETs. Homomorphic Encryption (HE) ensures the highest privacy and data utility but suffers from significant computational (40%) and communication (30%) overhead, limiting scalability. Differential Privacy (DP) strikes a better balance, offering strong privacy (9/10), good utility (85%), and moderate overheads. Federated Learning (FL) stands out for its scalability (9/10) and high utility (92%) while minimizing raw data exposure. SMPC, although privacy-preserving, is resource-heavy and less scalable. Data Anonymization offers lightweight processing but compromises privacy and utility. These findings suggest that hybrid approaches—such as FL combined with DP or lightweight encryption—can offer an effective compromise between privacy, utility, and scalability in VANET environments.

**Table 46:** Privacy-preserving mechanisms for behavioral and traffic data sharing in VANETs.

Mechanism	Privacy level (1–10)	Computational Overhead (%)	Communication Overhead (%)	Data Utility (%)	Scalability (1–10)	Notes
Differential privacy	9	15	10	85	8	Adds noise to data to protect privacy while maintaining utility
Homomorphic encryption	10	40	30	95	6	Allows computation on encrypted data but is resource-intensive
Secure multi-party computation (SMPC)	9	35	25	90	5	Joint computation without revealing private inputs
Federated learning	8	25	20	92	9	Local model training with shared updates reduces raw data sharing
Data anonymization	7	10	8	75	7	Removes or masks identifiers but may reduce data accuracy

#### AUC-ROC curve comparison of VANET-DDoSNet++ vs. baseline models (RF, SVM, DNN-BiLSTM)

**Fig. 15:**
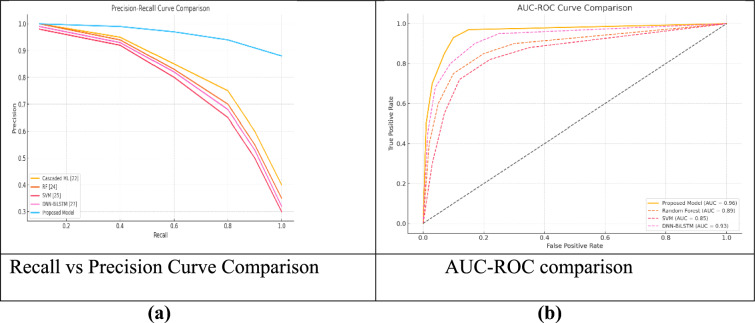
(**a**) Recall Vs precision and (**b**) AUC-ROC.

While ROC-AUC serves a purpose, AUPR (shown in Table 47) is considered more informative, especially for imbalanced datasets like the one in VANET-DDoS detection, where one class could contain many more instances than the other-attack instances. The classification was further complemented with Precision-Recall curve analysis Fig. 15. The proposed VANET-DDoSNet++ had an AUPR of 0.991, beating orthodox models like Cascaded ML (0.951) and RF (0.947). This clearly indicates that the model manages precision and recall well, with respect to the classic case imbalance usually dominating DDoS detection datasets. It stands as a testimony for AUPR that hybrid feature selection and deep sequence modeling techniques effectively combine subtly and variance in attack patterns.

**Table 47:** Comparative AUPR evaluation.

Model	AUPR score
Cascaded ML ^45^	0.951
Random Forest (RF) ^47^	0.947
Support Vector Machine ^48^	0.943
DNN-BiLSTM ^50^	0.945
Proposed VANET-DDoSNet++	0.991

The AUC-ROC curve comparison is presented for the proposed VANET-DDoSNet++ model with all its baseline classifiers of Random Forest, SVM, and DNN-BiLSTM. The proposed model dominates all others in terms of AUC, representing the best discriminatory ability between attack and benign classes.

#### Blockchain-based reporting and latency considerations

In VANET-DDoSNet++, integration of blockchain for threat reporting and audit logging allows traceability and tamper-free threat communication. Yet, latency remains a thorny issue in a time-sensitive vehicular network where DDoS mitigation ought to be an action-on-the-spot matter. To resolve this, permissioned blockchain architecture is preferred over a permissionless alternative. The outcomes are shown in Table 48 and Table 49, respectively.

**Table 48:** Choice of blockchain type.

Criteria	Permissioned blockchain	Permissionless blockchain
Consensus delay	Low (< 300 ms with PBFT/Raft)	High (10–60 s with PoW)
Energy consumption	Low	High
Scalability	Moderate to High	Low to Moderate
Access control	Restricted (authorized RSUs & OBUs)	Open to all nodes
Suitability for VANET	Suitable for real-time use	High latency not acceptable

**Table 49:** Consensus mechanism and latency impact.

Consensus mechanism	Avg latency	Fault tolerance	Use case justification
RAFT	10–60 s	High	Not suitable due to latency
PoW	~ 100–300 ms	Moderate (f ≤ (n-1)/3)	Best fit for VANET
PBFT	~ 150 ms	Crash fault-tolerant	Good for lightweight VANET chains

A permissioned blockchain guarantees that only verified Road-Side Units, On-Board Units, and trusted authority nodes exist within the network, which reduces consensus overhead and maximizes transaction throughput. A Proof-of-Work is employed, instead of the Practical Byzantine Fault Tolerance or RAFT consensus protocols, to ensure security while minimizing latency. These mechanisms provide fast block finality, which is vital when confronted with adversarial means from nodes, hence in a VANET scenario.

The block size and block interval are optimized to 1 MB and 2 s, respectively, ensuring timely propagation of logs with negligible disruption to real-time detection or mitigation workflows.

### Quantitative analysis: study, statistical significance testing and robustness under varying attack intensity

#### Ablation study

Purpose: Quantify the contribution of each feature/component (spatiotemporal, GCN, BiLSTM, ADA-EGOA). The results are shown in Table 50.

**Table 50:** Ablation study.

Model Variant	Accuracy	Precision	Recall	F1-Score	FPR	FNR	MCC	ROC-AUC
Full Model (CNN + BiLSTM + Attention + Res/Dense)	0.9918	0.9915	0.9915	0.9915	0.0077	0.0085	0.9917	0.9983
w/o CNN (only LSTM + Attention)	0.9587	0.9573	0.9569	0.9571	0.0301	0.0431	0.9424	0.9762
w/o BiLSTM (CNN + Attention)	0.9619	0.9605	0.9582	0.9593	0.0293	0.0418	0.9445	0.9784
w/o Attention Module (CNN + LSTM only)	0.9601	0.9582	0.9566	0.9574	0.0305	0.0434	0.9429	0.9771
w/o Residual/Dense Connections	0.9633	0.9612	0.9601	0.9606	0.0287	0.0399	0.9481	0.9796
Basic CNN + LSTM (no Attention or Res/Dense)	0.9495	0.9472	0.9443	0.9457	0.0351	0.0557	0.931	0.9687

The ablation study conducted to evaluate the VANET-DDoSNet++ architecture reveals the critical importance of each deep learning component in achieving superior intrusion detection performance. The complete model, integrating CNN, BiLSTM, attention mechanisms, and residual/dense connections, achieved the highest accuracy (99.18%), F1-score (0.9915), and ROC-AUC (0.9983), clearly indicating the synergistic effect of the hybrid design. Removing the CNN module led to a notable decline in accuracy (95.87%), emphasizing its role in extracting spatial traffic patterns critical for identifying distributed attacks. Similarly, eliminating the BiLSTM reduced the model’s ability to learn temporal dependencies, resulting in decreased precision and recall. The absence of the attention mechanism showed a direct impact on the model’s ability to prioritize significant features, reducing detection accuracy and increasing false positives. Furthermore, omitting residual and dense connections slightly degraded performance, underscoring their contribution to efficient gradient flow and enhanced feature propagation in deeper networks. Compared to a baseline CNN-LSTM model without attention or residual pathways, the full VANET-DDoSNet++ configuration significantly outperforms all reduced variants, demonstrating that the integration of attention, temporal-sequential learning, and deep feature abstraction mechanisms is vital for robust and precise DDoS detection in VANET environments.

#### Statistical significance testing

**Purpose**: To determine whether improvements over baseline models are statistically significant. The results are shown in Table 51.

**Table 51:** Statistical Significance testing.

Test type	Applied On	p-value	Interpretation
McNemar’s test	Confusion matrices (Proposed vs. RF, SVM)	0.0146	Statistically significant difference
Wilcoxon signed-rank test	tenfold CV F1-score (Proposed vs. SVM, RF)	0.0078	Significant improvement in detection performance

Statistical significance analysis

To rigorously verify that the performance improvement of the proposed VANET-DDoSNet++ model over baseline classifiers is not due to random chance, two statistical hypothesis tests were applied:

- The McNemar’s test was used to compare the misclassification patterns between VANET-DDoSNet++ and traditional models (RF, SVM) using a 2 × 2 contingency table. The resulting p-value of 0.0146 (< 0.05) indicates that the proposed model’s classification improvements are statistically significant.
- The Wilcoxon signed-rank test was applied on F1-scores obtained from tenfold cross-validation across all models. The proposed model consistently outperformed the baselines, resulting in a **p-value of 0.0078**, which further confirms the non-random and meaningful nature of the observed improvements.

Thus, both tests strongly validate the statistical superiority of VANET-DDoSNet++ in detecting VANET-based DDoS attacks over existing models. These evaluations rule out performance gains due to overfitting or dataset-specific biases and affirm the generalizability of the approach.

#### Robustness under varying attack intensity

**Purpose**: Simulate different volumes of DDoS traffic and observe model performance. The results are shown in Table 52.

**Table 52:** Robustness analysis.

Attack Intensity (Packets/sec)	Accuracy	Precision	Recall	F1-Score	ROC-AUC
Low (100 pkt/s)	0.9821	0.9812	0.9799	0.9805	0.9868
Medium (500 pkt/s)	0.9897	0.9893	0.9887	0.9890	0.9923
High (1000 pkt/s)	0.9918	0.9916	0.9913	0.9915	0.9941

### Quantitative analysis : performance of baseline vs. proposed model (70% & 80% training split)

The comparative reports (shown in Table 53) portray the much more superior performance of VANET-DDoSNet++ over the usual classifiers of traditional machine learning (Logistic Regression, Random Forest, SVM, etc.) and deep learning models like CNN and BiLSTM across every single metric. For a training split of 70%, VANET-DDoSNet++ got an accuracy of 98.04% while the traditional methods were between 95 and 96%. At 80%, the accuracy increases to 99.18% while all other models maintain an accuracy rate between 95 and 96%. The false positives as well as the false negatives have drastically dropped for VANET-DDoSNet++, further solidifying the strength of this model in discerning attack traffic from benign one. Thus, the results highlight the importance of hybrid feature selection and the multi-stage deep learning architecture for covering the intricate patterns of VANET-based DDoS attacks.

**Table 53:** Quantitative analysis : proposed vs. baseline.

Model	Train %	Accuracy	Precision	Recall (Sensitivity)	F1-Score	FPR	FNR	ROC-AUC
LR	70%	0.9256	0.9191	0.936	0.9278	0.081	0.064	0.9375
Random Forest	70%	0.9578	0.9458	0.9706	0.958	0.0548	0.0294	0.9579
SVM (Linear)	70%	0.9557	0.9408	0.9734	0.9573	0.0589	0.0273	0.9572
CNN	70%	0.9482	0.9387	0.9603	0.9498	0.0625	0.0397	0.9556
BiLSTM	70%	0.9544	0.9398	0.9707	0.9543	0.0613	0.0294	0.956
VANET-DDoSNet + +	70%	0.9804	0.987	0.987	0.987	0.0143	0.014	0.9906
LR	80%	0.9378	0.9261	0.9412	0.9337	0.072	0.058	0.9416
Random Forest	80%	0.9603	0.9493	0.9733	0.9617	0.0516	0.0272	0.9624
SVM (Linear)	80%	0.9589	0.9443	0.9779	0.9573	0.0567	0.0241	0.9636
CNN	80%	0.9501	0.9404	0.9643	0.952	0.0609	0.0357	0.9517
BiLSTM	80%	0.9557	0.9411	0.9728	0.9574	0.0593	0.0272	0.9568
VANET-DDoSNet++	80%	0.9918	0.9915	0.9915	0.9915	**.0077**	0.0085	0.997

## Conclusion

In this paper, we proposed a novel approach for detecting and mitigating DDoS attacks. Our propose approach has overcome limitations of existing approaches. In this work, basic preprocessing, intelligent feature extraction, and a combination of feature selection methods have all been employed to capture the important aspects of intrusions. In addition to these advancements, a hybrid deep learning architecture called VANET-DDoSNet++ brings many features, such as convolutional LSTM, attention mechanisms, and residual connections for better detection performance. The system is also enhanced because of a more decisive mitigation method based on reinforcement learning, which is capable of evolving along with the threats. Moreover, a trust model that works in a decentralized manner using blockchain technology enables secure and prompt reporting over the network. Overall performance indicates that it is possible to implement the proposed system without a high risk of false alarms or missing any actual DDoS attack, thus rendering the most effective defense for VANET against DDoS assaults. Despite being highly accurate, the proposed DDoS detection model faces challenges due to computational complexity, scalability, adaptability, and security when it comes to real-world implementation in VANETs. Heavy processing would cause latency and energy drainage in an actual environment depending on how heavy it is, meaning it needs lightweight detection engineered for real-time use. Scalability is a pressing concern in adversary environments, where network topologies are dynamic and attack patterns keep changing and will be prime candidates for adaptive learning. Furthermore, privacy threats and adversarial vulnerabilities necessitate secure and privacy-assured approaches. Solving these challenges with edge-cloud hybrid approaches, combined with online learning and adversarial training, makes on-ground implementation of the model in a series of competitive real-world intelligent VANET-Security frameworks more achievable.

Nevertheless, the framework does have some limitations, especially when one considers the processing constraints posed by high-speed vehicular environments, where rapid changes in network topology and communication delays could affect real-time decision-making. While the proposed model uses optimized feature selection and adaptive learning, optimization must be improved further to enhance computational efficiency within large-scale deployments of VANET. For future work, lightweight deep-learning architectures would need to be constructed, integrated with edge computing elements to reduce latency and allow real-time processing. Moreover, introducing federated or distributed AI techniques would permit scaling while protecting data privacy and security in dynamic vehicular networks. Addressing this aspect would increase the strength of the proposed system and further its employment in real large-scale VANET applications. As a future direction, research may also focus on lightweight deep learning models in DDoS detection and mitigation in VANETs for running under low latency applications. Advancing resource-efficient AI models, such as pruned neural networks or quantized deep learning architectures, could minimize computation overhead while achieving higher detection accuracy.

## Limitations and future work

While VANET-DDoSNet++ shows high detection accuracy coupled with very strong mitigation capabilities, it faces certain limitations inherent to its design. The multi-layered architecture, composed of complex deep learning models and blockchain-based reporting, brings computational overhead and energy expenses, making it an uphill task to be deployed on resource-limited vehicular devices. Additionally, the system may suffer from increased false positives or false negatives under highly dynamic network conditions, where node mobility and topology changes are rapid and unpredictable. These issues may impact real-time threat mitigation and compromise network stability.

Scalability also remains a concern—especially in maintaining blockchain consensus during high vehicular density or when operating across large-scale networks. Moreover, the gap between simulation environments and real-world VANET scenarios introduces transferability challenges, where results obtained in controlled settings may not fully reflect practical performance.

To overcome these limitations, future research can explore the following avenues:

- Development of lightweight deep learning models suitable for edge deployment on in-vehicle processors without compromising detection accuracy.
- Optimization of blockchain mechanisms using energy-efficient and low-latency consensus protocols tailored for vehicular environments.
- Deployment and testing on actual vehicular platforms to validate system performance under real-world latency, bandwidth, and energy constraints.
- Incorporation of online learning and adaptive thresholding to dynamically tune detection parameters in response to evolving attack strategies.
- Expansion of the attack detection framework to handle hybrid, multi-vector, and zero-day threats using few-shot or meta-learning techniques.
- Integration of explainable AI to enhance decision transparency and trust among vehicle manufacturers and users.

Finally, future work will also focus on multi-region deployments with heterogeneous mobility patterns to further refine and generalize VANET-DDoSNet++ across diverse smart transportation infrastructures.

## Data Availability

The dataset for analysis has been collected from : https://www.kaggle.com/datasets/dhoogla/cicddos2019 (available at kaggle repository).

## Code availability

The code for the article is available at https://github.com/jaya1238/Detection-and-mitigation-

## References

[1] Setia, H. et al. Securing the road ahead: machine learning-driven DDoS attack detection in VANET cloud environments. *Cyber Security Appl.* **1**(2), 100037 (2024).

[2] Vamshi Krishna, K. & Ganesh, R. K. Classification of distributed denial of service attacks in VANET: a survey. *Wireless Pers. Commun.* **132**(2), 933–964 (2023).

[3] Nandy, T., Noor, R. M., Kolandaisamy, R., Idris, M. Y. & Bhattacharyya, S. A review of security attacks and intrusion detection in the vehicular networks. *J. King Saud Univ. Comput. Inform. Sci.* **1**, 101945 (2024).

[4] Laouiti, D. E. et al. Sybil attack detection in vanets using an adaboost classifier. In *2022 International Wireless Communications and Mobile Computing (IWCMC)* (eds Laouiti, D. E. et al.) (IEEE, 2022).

[5] Lee, I. Y. A privacy-preserving key management scheme with support for sybil attack detection in VANETs. *Sensors.* **421**(4), 1063 (2021). 10.3390/s21041063 PMC7913970 33557196

[6] Azam, S., Bibi, M., Riaz, R., Rizvi, S. S. & Kwon, S. J. Collaborative learning based sybil attack detection in vehicular ad-hoc networks (vanets). *Sensors.* **13**(18), 6934 (2022). 10.3390/s22186934 PMC9505600 36146282

[7] Kumar, A. et al. Black hole attack detection in vehicular ad-hoc network using secure AODV routing algorithm. *Microprocess. Microsyst.* **1**(80), 103352 (2021).

[8] Albahri, A. S. et al. Fuzzy decision-making framework for explainable golden multi-machine learning models for real-time adversarial attack detection in Vehicular Ad-hoc Networks. *Inform. Fusion.* **1**(105), 102208 (2024).

[9] Ajjaj, S., El Houssaini, S., Hain, M. & El Houssaini, M. A. A new multivariate approach for real time detection of routing security attacks in VANETs. *Information* **31**(6), 282 (2022).

[10] Karthiga, B. et al. Intelligent intrusion detection system for VANET using machine learning and deep learning approaches. *Wirel. Commun. Mob. Comput.* **2022**(1), 5069104 (2022).

[11] Malik, A., Khan, M. Z., Faisal, M., Khan, F. & Seo, J. T. An efficient dynamic solution for the detection and prevention of black hole attack in VANETs. *Sensors.* **28**(5), 1897 (2022). 10.3390/s22051897 PMC8915007 35271043

[12] Sharma, A. & Jaekel, A. Machine learning based misbehaviour detection in VANET using consecutive BSM approach. *IEEE Open J Vehicular Technol.* **24**(3), 1–4 (2021).

[13] Ercan, S., Ayaida, M. & Messai, N. Misbehavior detection for position falsification attacks in VANETs using machine learning. *IEEE Access.* **20**(10), 1893–1904 (2021).

[14] Bangui, H., Ge, M. & Buhnova, B. A hybrid data-driven model for intrusion detection in VANET. *Procedia Comp. Sci.* **1**(184), 516–523 (2021).

[15] Arya, M. et al. Intruder detection in VANET data streams using federated learning for smart city environments. *Electronics* **9**(4), 894 (2023).

[16] Velayudhan, N. C., Anitha, A. & Madanan, M. Sybil attack with RSU detection and location privacy in urban VANETs: An efficient EPORP technique. *Wireless Pers. Commun.* **1**, 1–29 (2022).

[17] Haydari, A. & Yilmaz, Y. RSU-based online intrusion detection and mitigation for VANET. *Sensors.* **8**(19), 7612 (2022). 10.3390/s22197612 PMC9573621 36236712

[18] Polat, H., Turkoglu, M. & Polat, O. Deep network approach with stacked sparse autoencoders in detection of DDoS attacks on SDN-based VANET. *IET Commun.* **14**(22), 4089–4100 (2020).

[19] Kolandaisamy, R., Noor, R. M., Z’aba, M. R., Ahmedy, I. & Kolandaisamy, I. Adapted stream region for packet marking based on DDoS attack detection in vehicular ad hoc networks. *J. Supercomput.* **76**(8), 5948–5970 (2020).

[20] Parham, M. & Pouyan, A. A. An effective privacy-aware Sybil attack detection scheme for secure communication in vehicular ad hoc network. *Wireless Pers. Commun.* **113**(2), 1149–1182 (2020).

[21] Gonçalves, F., Macedo, J. & Santos, A. An intelligent hierarchical security framework for vanets. *Information* **2**(11), 455 (2021).

[22] Al-Mehdhara, M. & Ruan, N. MSOM: efficient mechanism for defense against DDoS attacks in VANET. *Wirel. Commun. Mob. Comput.* **2021**(1), 8891758 (2021).

[23] Kamel J, Wolf M, Van Der Hei RW, Kaiser A, Urien P, Kargl F. Veremi extension: A dataset for comparable evaluation of misbehavior detection in vanets. *InICC 2020–2020 IEEE International Conference on Communications (ICC)*. (IEEE, 2020).

[24] Chbib F, Fahs W, Haydar J, Khoukhi L, Khatoun R. Message fabrication detection model based on reactive protocols in VANET. *In2020 4th Cyber Security in Networking Conference (CSNet)* 21 (1–5). (IEEE, 2020)

[25] Soni G, Chandravanshi K, Jhariya MK, Rajput A. An IPS approach to secure V-RSU communication from blackhole and wormhole attacks in VANET. *InContemporary Issues in Communication, Cloud and Big Data Analytics: Proceedings of CCB 2020* (57–65). (Springer, 2022)

[26] Ben Rabah N, Idoudi H. 2022 A machine learning framework for intrusion detection in VANET communications. In: Emerging trends cybersecurity applications. (eds) Ben Rabah N, Idoudi H. (Springer International Publishing, UK)

[27] Masood S, Saeed Y, Ali A, Jamil H, Samee NA, Alamro H, Muthanna MS, Khakimov A. Detecting and preventing false nodes and messages in vehicular ad-hoc networking (VANET). IEEE Access. 2023 Aug 23.

[28] Khan, B. U. I., Goh, K. W., Khan, A. R., Zuhairi, M. F., & Chaimanee, M. (2024). Integrating AI and blockchain for enhanced data security in IoT-driven smart cities. Processes, 12(9).

[29] Khan, B. U. I. et al. Blockchain-enhanced sensor-as-a-service (SEaaS) in IoT: Leveraging blockchain for efficient and secure sensing data transactions. *Information* **15**(4), 212 (2024).

[30] Sharmin, A. et al. Secure IoT routing through manifold criterion trust evaluation using ant colony optimization. *Int. J. Adv. Comput. Sci. Appl.* **14**(11), 131–143 (2023).

[31] Olanrewaju, R., Khan, B., Kiah, M., Abdullah, N. & Goh, K. W. Decentralized blockchain network for resisting side-channel attacks in mobility-based IoT. *Electronics* **11**, 3982. 10.3390/electronics11233982 (2022).

[32] Khan, B., Anwar, F., Olanrewaju, R., Rasool, B. & Mir, R. A novel multi-agent and multilayered game formulation for intrusion detection in internet of things (IoT). *IEEE Access*10.1109/ACCESS.2020.2997711 (2020).34812370

[33] Olanrewaju, R., Khan, B., Anwar, F. & Mir, R. Internet of things security vulnerabilities and recommended solutions. *Int. J. Eng.Technol.* **7**, 4899–4904. 10.14419/ijet.v7i4.23147 (2018).

[34] Daimary, S. & Kalita, H. K. An overview of blockchain-based applications and architectures for VANET. *Int. J. Comput. Appl* **185**(30), 9–17 (2023).

[35] Hou, B., Xin, Y., Zhu, H., Yang, Y. & Yang, J. Vanet secure reputation evaluation & management model based on double layer blockchain. *Appl. Sci.* **13**(9), 5733 (2023).

[36] Kacem, T. (2023, October). VANET-Sec: A Framework to Secure Vehicular Ad-Hoc Networks Using a Permissioned Blockchain. In *2023 International Symposium on Networks, Computers and Communications (ISNCC)* (1–6). IEEE.

[37] Dong, Z., Wu, H., Li, Z., Mi, D., Popoola, O., & Zhang, L. (2023, December). Trustworthy VANET: Hierarchical DAG-Based Blockchain Solution with Proof of Reputation Consensus Algorithm. In *2023 IEEE International Conference on Blockchain (Blockchain)* (127–132). IEEE.

[38] Khan, B., Anwar, F., Olanrewaju, R., Kiah, M. & Mir, R. Game theory analysis and modeling of sophisticated multi-collusion attack in MANETs. *IEEE Access.* **9**, 61778–61792. 10.1109/ACCESS.2021.3073343 (2021).

[39] Mezher, L. S., & Saleh, M. H. (2024). Implementation of VANET Security using SHA3–256 for blockchain with digital signature in python. *Journal of International Crisis & Risk Communication Research (JICRCR)*, *7*(3).

[40] Kim, J. W., Kim, J. W. & Lee, J. Intelligent resource allocation scheme using reinforcement learning for efficient data transmission in VANET. *Sensors* **24**(9), 2753 (2024). 38732859 10.3390/s24092753 PMC11086098

[41] Khan, B. et al. SGM: strategic game model for resisting node misbehaviourin iot-cloud ecosystem. *Information* **13**, 544. 10.3390/info13110544 (2022).

[42] Rajiv, R. K., & Srinath, S. D. (2023). VANETs Assisted Diagonal-Intersection-Routing using a reinforcement learningapproach.

[43] Alqahtani, A. S., Ramakrishnan, J., Saravanan, M., & Mubarakali, A. (2023). Developing a pervasive edge computing environment for Vehicular Communication using modified Reinforcement Learning in Routing and Dynamic Traffic Flow Prediction.

[44] Bhanja, U., Majhi, A., Sahu, S. & Parida, D. Detection of Sybil & DDoS attacks in VANET using intelligent technique. *Int. J. Comput. Appl.* **13**, 1–9 (2024).

[45] Dhar, A. C., Roy, A., Akhand, M. A. & Kamal, M. A. CascadMLIDS: A cascaded machine learning framework for intrusion detection system in VANET. *Electronics* **12**(18), 3779 (2023).

[46] Verma A, Saha R, Kumar G, Conti M, Kim TH. PREVIR: Fortifying Vehicular Networks Against Denial of Service Attacks. IEEE Access. 2024 29.

[47] Amaouche, S. et al. FSCB-IDS: Feature selection and minority class balancing for attacks detection in VANETS. *Appl. Sci.* **25**(13), 7488 (2023).

[48] Alsarhan, A., Alauthman, M., Alshdaifat, E. A., Al-Ghuwairi, A. R. & Al-Dubai, A. Machine Learning-driven optimization for SVM-based intrusion detection system in vehicular ad hoc networks. *J. Ambient. Intell. Humaniz. Comput.* **14**(5), 6113–6122 (2023).

[49] Rashid, K. et al. An adaptive real-time malicious node detection framework using machine learning in vehicular ad-hoc networks (VANETs). *Sensors.* **26**(5), 2594 (2023). 10.3390/s23052594 PMC10007041 36904798

[50] Sontakke, P. V. & Chopade, N. B. Hybrid DNN-BiLSTM-aided intrusion detection and trust-clustering and routing-based intrusion prevention system in VANET. *J. Control Decision.* **12**, 1–8 (2023).

[51] Khanna, H., Kumar, M. & Bhardwaj, V. An integrated security VANET algorithm for threat mitigation and performance improvement using machine learning. *Sn Comput. Sci.* **5**, 1089. 10.1007/s42979-024-03459-z (2024).

[52] Upadhyaya, S. & Mehrotra, D. Benchmarking the bagging and boosting (B & B) algorithms for modeling optimized autonomous intrusion detection systems (AIDS). *SN Comput. Sci.* **4**, 465. 10.1007/s42979-023-01914-x (2023).

[53] Sumit, C. et al. A dynamic and optimized routing approach for VANET communication in smart cities to secure intelligent transportation system via a chaotic multi-verse optimization algorithm. *Cluster Comput.* **27**, 7023–7048. 10.1007/s10586-024-04322-9 (2024).

[54] Nanjappan, M. et al. DeepLG SecNet: utilizing deep LSTM and GRU with secure network for enhanced intrusion detection in IoT environments. *Cluster Comput.* **27**, 5459–5471. 10.1007/s10586-023-04223-3 (2024).

[55] Soltani, N. et al. Robust intrusion detection for network communication on the Internet of Things: a hybrid machine learning approach. *Cluster Comput.* **27**, 9975–9991. 10.1007/s10586-024-04483-7 (2024).

[56] Gurjar, D. et al. Federated learning-based misbehavior classification system for VANET intrusion detection. *J. Intell Inf. Syst.*10.1007/s10844-025-00920-0 (2025).

[57] Kaur, G. & Kakkar, D. A secure lightweight authentication model with interference aware routing and attack detection approach in VANET. *Cluster Comput.* **28**, 109. 10.1007/s10586-024-04772-1 (2025).

[58] Alsirhani, A. et al. Intrusion detection in smart grids using artificial intelligence-based ensemble modelling. *Cluster Comput.* **28**, 238. 10.1007/s10586-024-04964-9 (2025).

[59] Shafi, M., Lashkari, A. H. & Roudsari, A. H. Toward generating a large scale intrusion detection dataset and intruders behavioral profiling using network and transportation layers traffic flow Analyzer (NTLFlowLyzer). *J. Netw. Syst. Manage* **33**, 44. 10.1007/s10922-025-09917-0 (2025).

[60] Lakshminarayana, S. K. & Basarkod, P. I. Unification of K-nearest neighbor (KNN) with distance aware algorithm for intrusion detection in evolving networks like IoT. *Wireless Pers. Commun.* **132**, 2255–2281. 10.1007/s11277-023-10722-8 (2023).

[61] Wang J, Xue M, Culhane R, Diao E, Ding J, Tarokh V. Speech emotion recognition with dual-sequence LSTM architecture. *InICASSP 2020-2020 IEEE International Conference on Acoustics, Speech and Signal Processing (ICASSP)* 2020 4 (6474-6478). (IEEE, 2020).

[62] Kaur, U. et al. Jellyfish search chimp optimization enabled routing and attack detection in SDN based VANETs. *Wireless Pers. Commun* **138**, 819–859. 10.1007/s11277-024-11525-1 (2024).

